# Genomic and transcriptomic analysis of checkpoint blockade response in advanced non-small cell lung cancer

**DOI:** 10.1038/s41588-023-01355-5

**Published:** 2023-04-06

**Authors:** Arvind Ravi, Matthew D. Hellmann, Monica B. Arniella, Mark Holton, Samuel S. Freeman, Vivek Naranbhai, Chip Stewart, Ignaty Leshchiner, Jaegil Kim, Yo Akiyama, Aaron T. Griffin, Natalie I. Vokes, Mustafa Sakhi, Vashine Kamesan, Hira Rizvi, Biagio Ricciuti, Patrick M. Forde, Valsamo Anagnostou, Jonathan W. Riess, Don L. Gibbons, Nathan A. Pennell, Vamsidhar Velcheti, Subba R. Digumarthy, Mari Mino-Kenudson, Andrea Califano, John V. Heymach, Roy S. Herbst, Julie R. Brahmer, Kurt A. Schalper, Victor E. Velculescu, Brian S. Henick, Naiyer Rizvi, Pasi A. Jänne, Mark M. Awad, Andrew Chow, Benjamin D. Greenbaum, Marta Luksza, Alice T. Shaw, Jedd Wolchok, Nir Hacohen, Gad Getz, Justin F. Gainor

**Affiliations:** 1 Broad Institute of Massachusetts Institute of Technology (MIT) and Harvard, Cambridge, MA USA; 2 Lank Center for Genitourinary Oncology, Dana-Farber Cancer Institute, Boston, MA USA; 3 AstraZeneca, Oncology R&D, New York, NY USA; 4 Massachusetts General Hospital Cancer Center, Massachusetts General Hospital, Boston, MA USA; 5 Dana-Farber Cancer Institute, Boston, MA USA; 6 Center for the AIDS Programme for Research in South Africa, Durban, South Africa; 7 Center for Thoracic Cancers, Massachusetts General Hospital, Boston, MA USA; 8 GlaxoSmithKline, Waltham, MA USA; 9 Herbert Irving Comprehensive Cancer Center, Columbia University, New York, NY USA; 10 Department of Systems Biology, Columbia University Irving Medical Center, New York, NY USA; 11 Department of Thoracic and Head and Neck Oncology, MD Anderson Cancer Center, Houston, TX USA; 12 Department of Genomic Medicine, MD Anderson Cancer Center, Houston, TX USA; 13 Druckenmiller Center for Lung Cancer Research, Memorial Sloan Kettering Cancer Center, New York, NY USA; 14 Lowe Center for Thoracic Oncology, Dana-Farber Cancer Institute, Boston, MA USA; 15 Department of Medicine, Harvard Medical School, Boston, MA USA; 16 Bloomberg-Kimmel Institute for Cancer Immunotherapy, Johns Hopkins University School of Medicine, Baltimore, MD USA; 17 UC Davis Comprehensive Cancer Center, Sacramento, CA USA; 18 Department of Hematology and Medical Oncology, Cleveland Clinic, Cleveland, OH USA; 19 Department of Hematology and Oncology, NYU Langone Health, New York, NY USA; 20 Department of Radiology, Massachusetts General Hospital, Boston, MA USA; 21 Department of Pathology, Massachusetts General Hospital, Boston, MA USA; 22 Department of Biomedical Informatics, Columbia University, New York, NY USA; 23 Department of Biochemistry and Molecular Biophysics, Columbia University, New York, NY USA; 24 Department of Medicine, Vagelos College of Physicians and Surgeons, Columbia University, New York, NY USA; 25 J.P. Sulzberger Columbia Genome Center, New York, NY USA; 26 Yale Cancer Center, Yale School of Medicine, New Haven, CT USA; 27 Department of Pathology, Yale School of Medicine, New Haven, CT USA; 28 Synthekine, Inc., Menlo Park, CA USA; 29 Computational Oncology, Department of Epidemiology and Biostatistics, Memorial Sloan Kettering Cancer Center, New York, NY USA; 30 Physiology, Biophysics & Systems Biology, Weill Cornell Medicine, Weill Cornell Medical College, New York, NY USA; 31 Department of Oncological Sciences, Icahn School of Medicine at Mount Sinai, New York, NY USA; 32 Weill Cornell Medicine, New York, NY USA; 33 Center for Cancer Research, Massachusetts General Hospital, Boston, MA USA

**Keywords:** Non-small-cell lung cancer, Genome informatics, Cancer therapy

## Abstract

Anti-PD-1/PD-L1 agents have transformed the treatment landscape of advanced non-small cell lung cancer (NSCLC). To expand our understanding of the molecular features underlying response to checkpoint inhibitors in NSCLC, we describe here the first joint analysis of the Stand Up To Cancer-Mark Foundation cohort, a resource of whole exome and/or RNA sequencing from 393 patients with NSCLC treated with anti-PD-(L)1 therapy, along with matched clinical response annotation. We identify a number of associations between molecular features and outcome, including (1) favorable (for example, *ATM* altered) and unfavorable (for example, *TERT* amplified) genomic subgroups, (2) a prominent association between expression of inducible components of the immunoproteasome and response and (3) a dedifferentiated tumor-intrinsic subtype with enhanced response to checkpoint blockade. Taken together, results from this cohort demonstrate the complexity of biological determinants underlying immunotherapy outcomes and reinforce the discovery potential of integrative analysis within large, well-curated, cancer-specific cohorts.

## Abstract (web summary)

Genomic and transcriptomic analysis of 393 non-small cell lung cancer patients treated with checkpoint inhibitors identifies molecular features associated with response.

## Main

The introduction of PD-1/PD-L1 inhibitors in the management of advanced non-small cell lung cancer (NSCLC) has led to a major paradigm shift in the treatment of the disease. Following multiple studies demonstrating improved overall survival, these agents have garnered approval either alone^1–4^ or in combination with chemotherapy^5,6^ or CTLA4 blockade^7^. However, with responses observed in only one in five unselected patients^1–3^, improved predictors of response are needed to identify patients most likely to benefit.

Given that the potential for long-term disease control is only realized in a minority of patients, extensive effort has been dedicated to identifying biomarkers of response and resistance. The dominant biomarkers to date are PD-L1 protein expression on tumor cell membranes^4^ and tumor mutational burden (TMB)^8–10^, which may underlie the generation of neoantigens that can serve as targets for immune recognition and targeting.

While additional features have begun to emerge including potential roles for mutation clonality^11^, an inflamed microenvironment^12,13^ and alterations in individual genes such as *EGFR*^14,15^ and *STK11* (ref. ^16^), further identification and integration of relevant predictors have been hindered by the absence of large, multi-omic, NSCLC-specific patient cohorts.

Here we describe findings from the first integrative analysis of the Stand Up To Cancer-Mark Foundation (SU2C-MARK) NSCLC cohort, a dataset of 393 patients treated with checkpoint inhibitors in the advanced-stage setting. We performed whole exome sequencing (WES) and RNA sequencing (RNA-seq) along with detailed clinical response assessments, enabling the composite assessment of genomic and transcriptomic biomarkers of response and resistance. Collectively, these richly annotated data will be a resource to the field in furthering both the basic and applied investigation into the role of PD-1/PD-L1 agents in advanced NSCLC.

## Results

### Cohort description and mutation summary

**Fig. 1:**
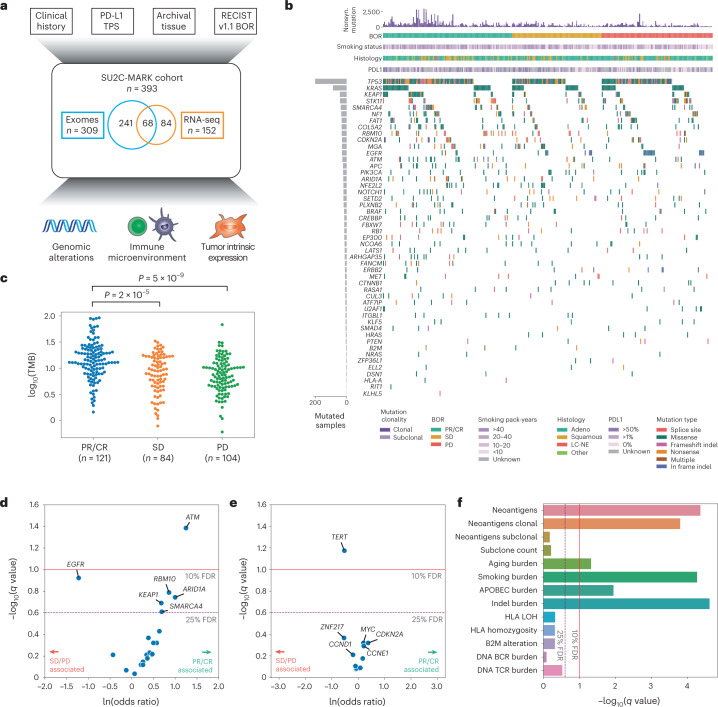
Overview of the SU2C-MARK cohort and initial genomic characterization. **a**, Overview of clinical and genomic data collected across the SU2C-MARK cohort (*n* = 393 patients). **b**, CoMut plot of SU2C-MARK cohort organized by response category. **c**, Log_10_ of the TMB as a function of response category. Significance was assessed via a two-sided Mann–Whitney *U* test. **d**, Volcano plot of logistic regression results for oncogenic mutations in known lung cancer drivers and binned BOR category comparing patients with a PR or CR to patients with SD or PD. *ATM* alterations reached significance (*q* < 0.1, Benjamini–Hochberg), while *EGFR*, *RBM10*, *ARID1A*, *KEAP1* and *SMARCA4* were all near significance (*q* < 0.25). **e**, Volcano plot of logistic regression results for gene-level copy number. Focal amplifications of *TERT* as well as the cytoband it is located on, 5p15.33 (Extended Data Fig. 3b), are associated with resistance to checkpoint blockade. **f**, Summary of exome-derived genomic features and logistic regression with response. Neoantigens were estimated using NetMHCpan-4.0 (ref. ^60^) following *HLA* allele identification with POLYSOLVER^61^. Subclone count was assessed via PhylogicNDT^62^. Aging, smoking and APOBEC burdens were calculated based on the mutation burden attributable to these processes (SBS5, SBS4 and SBS13, respectively) following mutational signature analysis (Extended Data Fig. 4 and Methods). HLA was estimated via LOHHLA^24^. B- and T-cell rearranged receptor abundance was estimated via MiXCR^27^. LOH, loss of heterozygosity; TMB, tumor mutation burden; PR, partial response; CR, complete response; SD, stable disease; PD, progressive disease.

We analyzed formalin-fixed paraffin-embedded (FFPE) tumor samples collected before receipt of checkpoint blockade (defined as the first line of therapy in which a patient received a PD-1/PD-L1 agent) from a total of 393 patients with advanced NSCLC across nine cancer centers (Table 1 and Fig. 1a). The majority of these patients were treated with single-agent therapy (81%), with additional subsets receiving combination therapy including either CTLA4 blockade (17%) or chemotherapy (1%). Both tumor and matched normal specimens (from blood, or in rare cases, adjacent normal tissue) underwent WES; for a subset of patients, tumor tissue was additionally profiled by whole transcriptome RNA-seq. After stringent quality control (Methods), a total of 309 WES and 152 RNA-seq specimens were included for analysis. The primary outcome was best overall response (BOR) determined by a dedicated review of clinical imaging and quantified using RECIST v1.1 criteria.

**Table 1:** Baseline clinical characteristics of the SU2C-MARK cohort

Patient characteristics (*n* = 393)	All patients, no. (%)
Age (years), median (range)	64 (29–90)
Sex	
Male	182 (46)
Female	207 (53)
Smoking status	
Never	46 (12)
Former	283 (72)
Current	60 (15)
Smoking pack-years	
0	47 (12)
1–10	46 (12)
11–20	50 (13)
21–40	125 (32)
>40	113 (29)
Histology	
Adenocarcinoma	286 (73)
Squamous	77 (20)
LCNE	9 (2)
Other	17 (4)
PD-L1 expression	
0%	56 (14)
≥1%	168 (43)
Prior lines of therapy	
0	143 (36)
1	150 (38)
≥2	96 (24)
Therapy	
PD-(L)1 only	317 (81)
PD-(L)1 + CTLA4	65 (17)
PD-(L)1 + chemotherapy	2 (1)
BOR	
CR/PR	142 (36)
SD	110 (28)
PD	132 (33)

The SU2C-MARK cohort consists of 393 patients with NSCLC treated with immune checkpoint blockade therapy in the advanced setting. BOR to the first line containing a PD-(L)1 agent was recorded.

**Extended Data Fig. 1:**
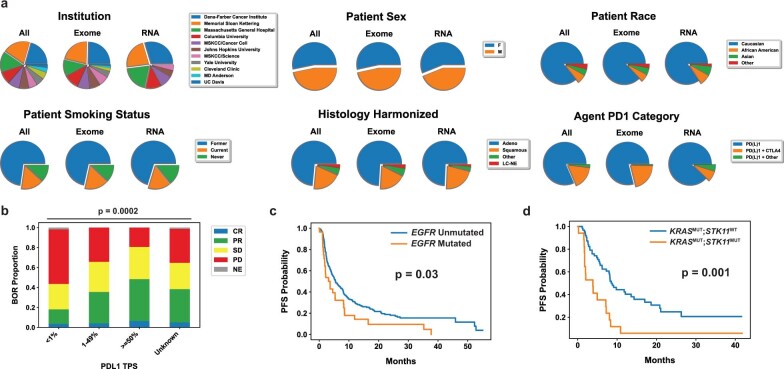
Extended SU2C-MARK cohort characterization and genomic predictor evaluation. **(a)** Distributions of clinical characteristics in the Stand Up To Cancer - Mark Foundation (SU2C-MARK) cohort. **(b)** Best overall response (BOR) distribution by PDL1 tumor proportion score (PDL1 TPS) category (significance assessed by two-sided Fisher’s exact test). CR = Complete Response, PR = Partial Response, SD = Stable Disease, PD = Progressive Disease, NE = Not Evaluable. **(c,d)** Kaplan-Meier curves for Progression-Free Survival (PFS) in *EGFR* mutated vs. unmutated patients (**c**) and *KRAS*/*STK11* comutated patients vs. *KRAS* mutant *STK11* umutated patients (**d**). Both *EGFR* mutated patients and *KRAS*/*STK11* comutated patients demonstrated decreased progression-free survival relative to their counterparts (p = 0.03 and p = 0.001, respectively, logrank test).

As is typical for patients with NSCLC, the SU2C-MARK cohort consisted predominantly of adenocarcinoma (73%) and squamous cell carcinoma (20%), with smaller contributions from large cell neuroendocrine (LCNE) carcinoma (2%) and other histologies (4%; Extended Data Fig. 1a). Among patients with annotated PD-L1 staining assessments (224/393 available, 43% missing), 25% had a tumor proportion score (TPS) of less than 1%, 33% had PD-L1 TPS 1–49%, and 42% had PD-L1 TPS ≥ 50%. As expected, higher PD-L1 TPS was associated with an increased response rate to checkpoint blockade (Extended Data Fig. 1b). Thus, our dataset reflected the histologic and biomarker compositions typically observed in unselected, real-world NSCLC cohorts^17,18^.

### Somatic alterations and PD-(L)1 blockade response in NSCLC

**Extended Data Fig. 2:**
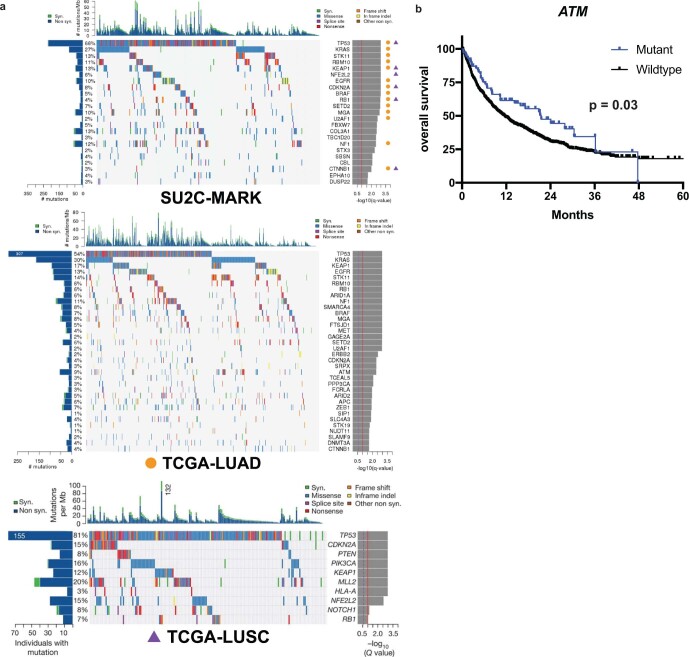
Extended analysis of mutated genes in the SU2C-MARK cohort and comparison to external cohorts. **(a)** Significant drivers identified independently in the SU2C-MARK cohort (left) as compared to TCGA Lung Adenocarcinoma (LUAD; right upper) and TCGA Lung Squamous Cell Carcinoma (LUSC; right lower). Of note, the SU2C-MARK cohort includes a mixture of frequent drivers observed in LUAD and LUSC, consistent with it representing pan-NSCLC histologies. **(b)** Kaplan-Meier curves comparing checkpoint blockade treated *ATM* mutant patients and *ATM* wildtype patients in the Memorial Sloan Kettering Cancer Center (MSKCC) Impact cohort. *ATM* mutated patients demonstrated improved survival compared to unmutated patients (p = 0.03, logrank test).

To better understand the relationship between mutational drivers and response, we assessed the prevalence of known drivers in lung cancer across our three response categories: partial or complete response (PR/CR), stable disease (SD) and progressive disease (PD; Fig. 1b and Extended Data Fig. 2a). Consistent with prior reports^8–10^, nonsynonymous TMB associated with response category (*P* = 6 × 10^−9^), with median TMB 14.0 mut/MB among those with PR/CR, compared to 9.0 mut/MB for SD, and 7.4 mut/MB for PD (Fig. 1c). Initial examination of the cohort was also consistent with previously observed driver associations^15,16,19^, such as *EGFR* alteration or *KRAS/STK11* comutation being a negative predictor of checkpoint blockade response (Extended Data Fig. 1c,d).

To facilitate a more comprehensive analysis, we performed logistic regression, testing the relationship between 49 known lung cancer drivers^20,21^ and response (that is, CR/PR versus SD/PD; Methods). In all, six genes achieved significance or near significance, defined as a false discovery rate (FDR) threshold of 10% or 25%, respectively (Fig. 1d). In this analysis, mutations in *ATM* appeared to be most favorable with respect to checkpoint blockade response (logistic regression FDR *q* = 0.04, OR = 3.5, CI_95%_ (1.5, 8.0)), while *EGFR* alterations were least favorable (*q* = 0.12, OR = 0.29, CI_95%_ (0.11, 0.79)). Given the strong association between *ATM* and response in our cohort, we tested this association in an independent cohort of patients with NSCLC treated with PD-(L)1 blockade and profiled by MSK-IMPACT^22^. In this external cohort, *ATM* alterations were associated with improved overall survival following checkpoint blockade (*P* = 0.03; Extended Data Fig. 2b). As this association was not seen at the cohort-wide level (*P* = 0.45), these results suggest a predictive rather than simply prognostic role for *ATM* alteration.

**Extended Data Fig. 3:**
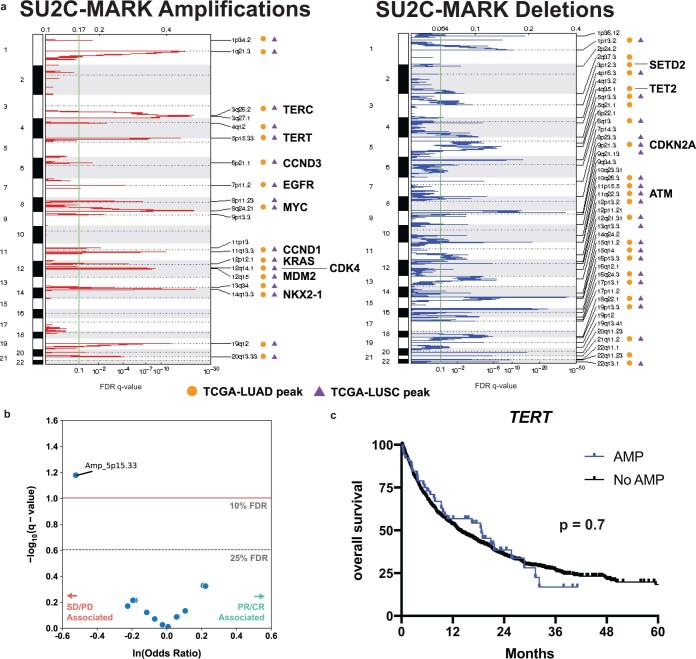
Extended analysis of somatic copy number alterations within the SU2C-MARK Cohort. **(a)** Somatic copy number alterations were analyzed using GISTIC2.0 (ref. ^87^) to identify significantly recurrent focal amplifications and deletions. Strong overlap between the events identified in the SU2C-MARK cohort and those previously described in TCGA LUAD and LUSC was observed. A subset of validated lung cancer drivers within regions of focal copy number alteration are annotated. **(b)** Volcano plot of logistic regression results for focal amplifications. Focal amplification of cytoband 5p15.33 (which contains *TERT*) is associated with resistance to checkpoint blockade. CR = Complete Response, PR = Partial Response, SD = Stable Disease, PD = Progressive Disease. **(c)** Kaplan-Meier curves comparing checkpoint blockade treated patients with and without *TERT* amplifications (AMP) in the Memorial Sloan Kettering Cancer Center (MSKCC) Impact cohort (p = 0.7, logrank test).

We next explored relationships between copy number alterations and response in the cohort (Extended Data Fig. 3a). Among focal events, only focal amplification of 5p15.33, the cytoband containing *TERT*, achieved significance, and was associated with reduced response to immunotherapy (*q* = 0.07, OR = 0.59, CI_95%_ (0.40, 0.87); Fig. 1e and Extended Data Fig. 3b). Of note, this association was not reproduced in the MSK-IMPACT cohort, which may be a function of the more limited sensitivity of amplifications in panel data (Extended Data Fig. 3c). Taken together, these results suggest that in addition to the aggregate metric of TMB, individual driver events may also define favorable and unfavorable NSCLC subsets for checkpoint blockade.

### Predicted neoantigens, antigen presentation and response

To better understand how the determinants of immune recognition in our cohort related to response, we calculated the neoantigen burden for each exome in the SU2C-MARK cohort (Methods). Total neoantigen burden was significantly associated with response (*q* = 4 × 10^−5^, OR = 8.8, CI_95%_ (4.2,19); Fig. 1f). As clonal neoantigens have been suggested to be more effective targets of immune recognition^11^, we additionally examined the role of clonal and subclonal neoantigen burden, along with total subclone count (Methods). Indeed, clonal neoantigen burden was also significantly associated with response (*q* = 2 × 10^−4^, OR = 5.4, CI_95%_ (2.7,11)), whereas neither subclonal neoantigen burden nor total subclone count was significant (*q* = 0.7 and *q* = 0.6, respectively; Fig. 1f).

**Extended Data Fig. 4:**
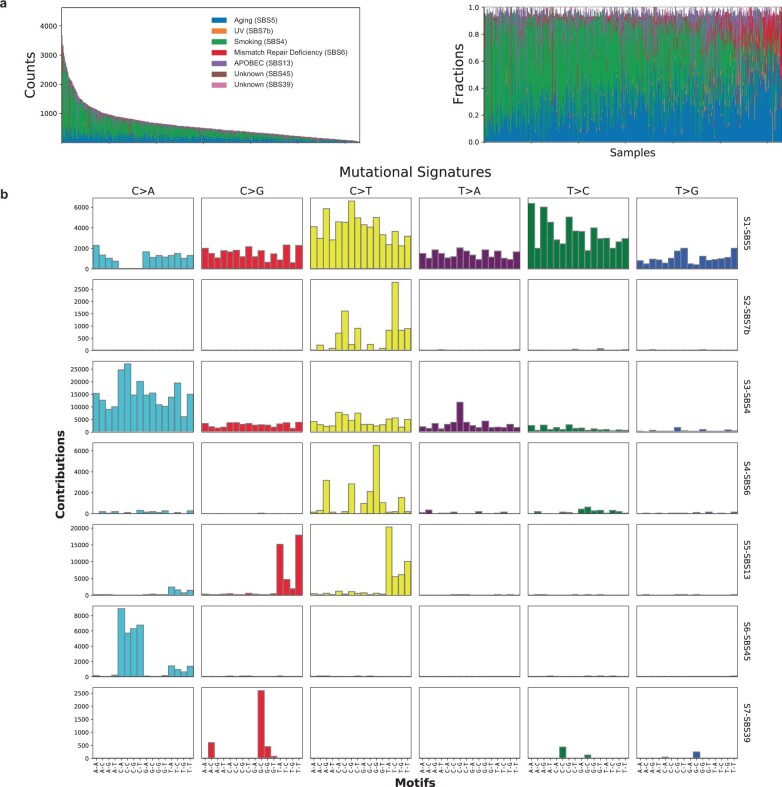
Mutation signature analysis in the SU2C-MARK and TCGA-LCNE cohorts. **(a)** Unsupervised mutational signature identification was performed using automatic relevance determination non-negative matrix factorization (ARD-NMF) on the combined SU2C-MARK and TCGA-LCNE cohorts. The TCGA-LCNE cohort comprises TCGA lung adenocarcinoma (LUAD), lung squamous cell carcinoma (LUSC), and published large cell neuroendocrine (LCNE)^63^ cohorts. Of the 7 signatures identified, the predominant signatures corresponded to COSMIC signatures for Aging (SBS5), Smoking (SBS4), and APOBEC (SBS13). Plots display mutational signatures identified in each sample based on mutation counts (left) as well as fraction of signature attributable mutations (right) with a shared color key for both plots. **(b)** Barplot of signature profiles demonstrating relative contribution from each 96-base context. Signatures were subsequently assigned to previously described COSMIC signatures based on cosine similarity^84^.

As different mutational processes may have different propensities for neoantigen generation, we also evaluated the mutation burden attributable to distinct mutational signatures (Extended Data Fig. 4a,b; Methods). Of the three dominant signatures, smoking was most strongly associated with response (*q* = 5 × 10^−5^), consistent with its association with clonal neoantigens, while aging (*q* = 0.05) and APOBEC (*q* = 0.01) were more weakly associated with response (Fig. 1f). We additionally observed a significant response association for indels (*q* = 2 × 10^−5^), which are suspected to be particularly immunogenic given their potential to generate new reading frames^11,23^.

Previous studies have suggested that compromised antigen presentation, due to loss of heterozygosity (LOH) at *HLA* loci^24^, decreased total unique *HLA* alleles^25^, or loss of *B2M*^26^ may enable immune evasion in certain cancer types. We did not observe an association of any of these factors measured before therapy and response in this cohort (Fig. 1f), potentially suggesting disease-specific variation in mechanisms of resistance.

To further assess for variation in immune infiltrate, we used MiXCR^27^ to identify B- and T-cell clonotypes from rearranged VDJ reads in our WES data (Methods). Of these subsets, T-cell receptor (TCR) burden was associated with response but did not reach statistical significance (*q* = 0.3). Thus, among our expanded set of exome-derived features, tumor-intrinsic markers reflective of TMB as well as clonal mutation burden emerged as top predictors of response.

### Transcriptional correlates of response

**Fig. 2:**
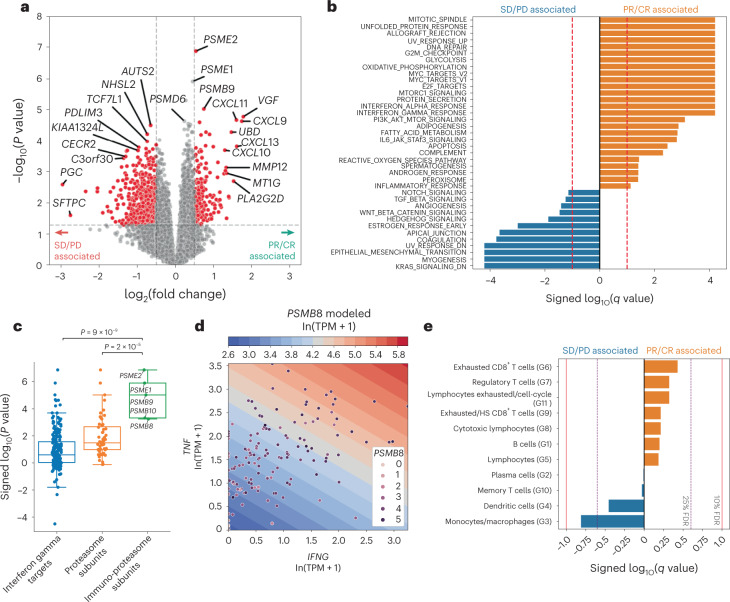
Transcriptomic features associated with response and resistance in the SU2C-MARK cohort. **a**, Volcano plot of limma voom results for top response-associated genes from RNA-seq samples in the SU2C-MARK cohort (*n* = 152 RNA samples). Nominal *P* values from two-sided significance testing are shown. Cutoffs of absolute log_2_(fold change) > 0.5 and *P* < 0.05 were used to identify significantly differentially expressed genes (red). **b**, Hallmark GSEA of response and resistance-associated pathways from limma voom. **c**, Dot plot of significance values for interferon-gamma (IFN-γ) targets (*n* = 198 genes), proteasome subunits (*n* = 56 genes) and immunoproteasome subunits (*n* = 5 genes). Boxplot overlay depicts the 25th percentile (minima), 50th percentile (center) and 75th percentile (maxima) of distribution with whiskers bounding points within 1.5× interquartile range (Q3–Q1) from each minimum and maximum. Immunoproteasome subunits as a set showed a greater association with response than IFN-γ targets and proteasome targets (*P* = 7 × 10^−9^ and *P* = 4 × 10^−6^, respectively, two-sided Mann–Whitney *U* test). **d**, Contour plot of a linear, 2D model predicting expression of representative immunoproteasome subunit *PSMB8* as a function of the inflammatory cytokines *IFNG* and *TNF*. Contour levels correspond to roughly 1.2-fold TPM increments in *PSMB8* expression. Patients with high expression of both *IFNG* and *TNF* demonstrated the highest *PSMB8* expression (*R*^2^ = 0.31). **e**, Logistic regression summary results for tumor-associated immune cell signatures derived from single-cell sequencing^37^.

**Extended Data Fig. 5:**
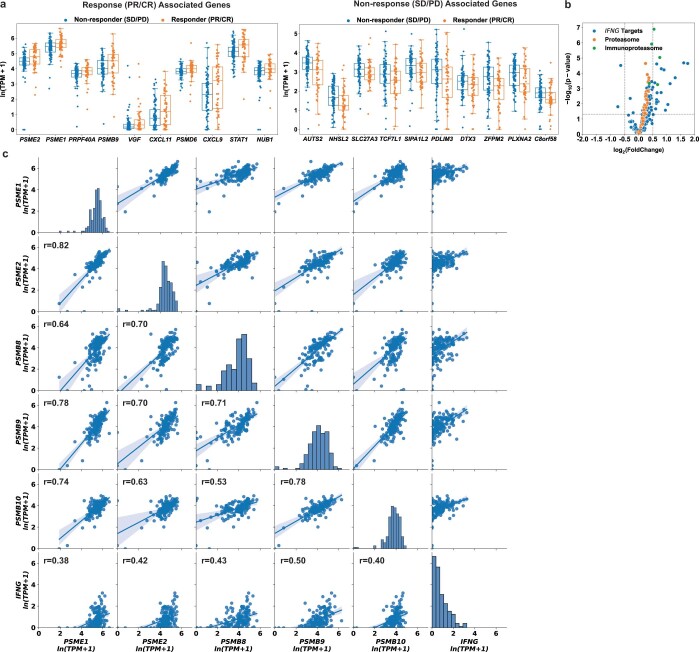
Extended response and resistance associated genes and signatures in the SU2C-MARK Cohort. **(a)** Expression of top 10 significant protein-coding transcripts associated with response (PR/CR, left; N = 52 RNA samples) and nonresponse (SD/PD, right; N = 84 RNA samples). Boxplot overlay depicts 25th percentile (minima), 50th percentile (center), and 75th percentile (maxima) of distribution with whiskers bounding points within 1.5 X interquartile range (Q3–Q1) from each minimum and maximum. PR = Partial Response, CR = Complete Response, SD = Stable Disease, PD = Progressive Disease, TPM = transcripts per million **(b)** Volcano plot for Limma results for cohort wide analysis subsetted to Interferon Targets, Proteasome Subunits, and Immunoproteasome Subunits. **(c)** Scatterplots comparing 5 inducible components of the immunoproteasome against each other as well as *IFNG*. Regression line and bootstrapped 95% confidence interval are displayed.

We next focused on the identification of transcriptional predictors of response. Using limma voom^28^, we performed a genome-wide analysis of differentially expressed genes between responders (PR/CR) and nonresponders (SD/PD; Fig. 2a and Methods). Initial assessment of these results identified three related genes that achieved cohort-wide significance (*p*_adj_ < 0.05; Methods): *PSME1*, *PSME2* and *PSMB9*. These genes are notable for their prominent role in the function of the immunoproteasome (further described below), a noncanonical peptide processing complex thought to promote differential and enhanced antigen presentation in the setting of proinflammatory cytokines^29^. Examination of the broader collection of genes achieving nominal significance (nominal *P* < 0.05) revealed additional interferon-gamma (IFN-γ)-induced transcripts including *TAP1* (a cytosolic peptide transporter in the antigen presentation pathway) and *CD274* (which encodes PD-L1), inflammatory chemokines such as *CXCL9, CXCL10* and *CXCL11*, and lymphocyte receptor genes (for example, *CD3D* and *CD7*), potentially surrogates for immune infiltration (Fig. 2a and Extended Data Fig. 5a). Top genes associated with nonresponse appear to span both developmental and immune-related pathways. *AUTS2* and *TCF7L1*, interacting transcription factors within the Wnt/B-catenin signaling axis, are postulated to have roles in both stem cell^30^ and immune signaling^31^. Another nonresponse-associated gene, *PDLIM3*, is a member of a protein family thought to negatively regulate NF-κB-mediated inflammatory responses^32^. *KALRN*, a guanine nucleotide exchange factor expressed in stromal and myeloid cells, has been associated with inflammation in the context of atherogenesis (Extended Data Fig. 5a).

To systematically identify differentially expressed pathways, we performed gene set enrichment analysis (GSEA) using the Hallmark Gene Sets^33^ (Fig. 2b). Top response-associated pathways included ALLOGRAFT_REJECTION, INTERFERON_GAMMA_RESPONSE and DNA_REPAIR, which has previously been observed as a predictor of checkpoint blockade response in urothelial carcinoma^34,35^. Pathways associated with resistance were diverse, with EPITHELIAL_MESENCHYMAL_TRANSITION, WNT_BETA_CATENIN_SIGNALING and TGF_BETA_SIGNALING gene sets all significantly associated with nonresponse (Fig. 2b). Taken together, these top genes and gene sets from bulk RNA-seq suggest the relevance of both immune and nonimmune components to the biology of checkpoint blockade.

### Immunoproteasome expression and response

Given the remarkable convergence of all three genes (*PSME1*, *PSME2* and *PSMB9*) on components of the proteasome/immunoproteasome system responsible for peptide generation, we expanded our exploration of genes specific to this antigen presentation pathway. Notably, *PSME1* and *PSME2* encode for the IFN-γ inducible PA28ɑβ complex that binds and enhances peptide processivity of both the constitutive and immunoproteasome. *PSMB9* (*LMP2*) encodes the β1i IFN-γ inducible subunit that together with β2i (*PSMB10*) and β5i (*PSMB8*) represent the three inducible subunits whose incorporation transforms the constitutive proteasome into a specialized immunoproteasome with distinct peptide cleavage patterns^29^. Hence, as all the inducible components of this complex (*PSMB8*, *PSMB9*, *PSMB10*, *PSME1* and *PSME2*) are known to be downstream of IFN-γ, which itself was nominally associated with response in our analysis (*IFNG* *P* = 0.001; log_2_ fold change 1.1), we evaluated the response association of these components alongside canonical IFN-γ targets (HALLMARK_INTERFERON_GAMMA_RESPONSE) as well as a comprehensive list of proteasome components (GOCC_PROTEASOME_COMPLEX; Fig. 2c). Notably, immunoproteasome components were enriched in terms of the significance of association with response relative to both IFN-γ targets more broadly, as well as all proteasome components (*P* = 9 × 10^−9^ and *P* = 2 × 10^−5^, respectively; Fig. 2c and Extended Data Fig. 5b).

Although the inducible subunits of the immunoproteasome were highly correlated with one another, increases in their expression could only partly be explained by elevated levels of IFN-γ (Extended Data Fig. 5c). Given that experimental evidence suggests they may also be induced by tumor necrosis factor-α (TNF-α)^36^, we evaluated whether higher levels of *TNF* may also contribute to upregulation of these components. Indeed, a linear combination of *IFNG* and *TNF* demonstrated improved model fit for immunoproteasome subunit expression (*R*^2^ = 0.31 for the combined model compared to 0.19 for the univariate model; Fig. 2d). Thus, immunoproteasome subunits appear to be singularly important predictors of response—even among the broader class of IFN-γ-induced transcripts—perhaps owing to their role as integrators of multiple cytokine cascades, enabling more efficient generation of peptide epitopes for HLA-I presentation.

### Immune subset signatures

Given that both individual gene and pathway level analysis highlighted key roles for immune signaling, we aimed to better delineate discrete immune cell subsets in our bulk transcriptome data using previously identified signatures derived from single-cell RNA data^37^ (Methods). Of the 11 signatures we evaluated, exhausted CD8^+^ T-cells showed the strongest positive association with response, while the monocyte/macrophage and dendritic cell signatures were most strongly associated with resistance (Fig. 2e).

**Extended Data Fig. 6:**
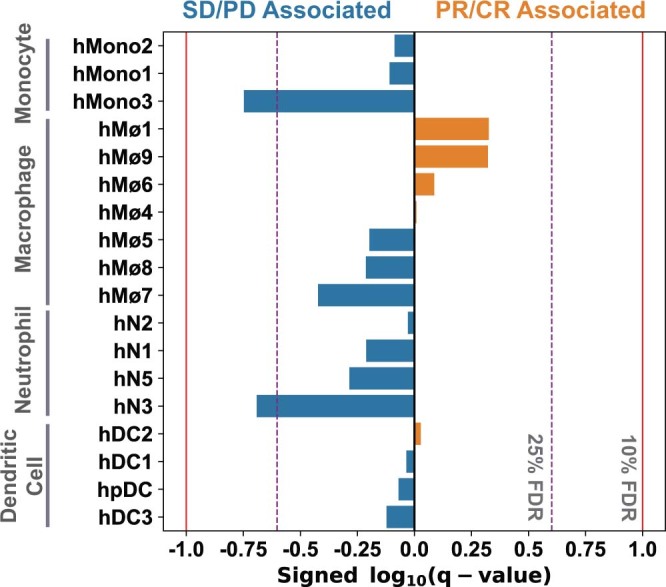
Significance testing of single cell profiling derived myeloid subsets and checkpoint blockade response in the SU2C-MARK Cohort. Logistic regression significance values for myeloid cell signatures derived from single cell profiling^40^ (Benjamini–Hochberg *q*-value). hMono3 and hN3 were classified as near-significant (*q* < 0.25) in their association with nonresponse. PR = Partial Response, CR = Complete Response, SD = Stable Disease, PD = Progressive Disease.

As a growing body of work suggests that distinct myeloid subsets may have differing roles in antitumor immunity^38,39^, we investigated more specific subsignatures related to these cell types. Using a marker list derived from a comprehensive single-cell RNA-seq study of infiltrating myeloid cells in human and mouse lung cancers^40^, we identified the hMono3 and hN3 subtypes as being particularly associated with resistance to checkpoint blockade (Extended Data Fig. 6). Notably, the hMono3 subtype is characterized by high expression of S100A8, a cytokine-like protein that can drive the accumulation of myeloid-derived suppressor cells^41^. The neutrophil hN3 subtype is defined by high expression of CXCR2, which has been shown to inhibit CD8 T-cell activation within the lung cancer microenvironment^42^. Thus, our focused analysis of immune subsets identified plausible mechanistic connections between myeloid infiltration and decreased response to checkpoint blockade.

### Microenvironmental (M) expression signatures

**Fig. 3:**
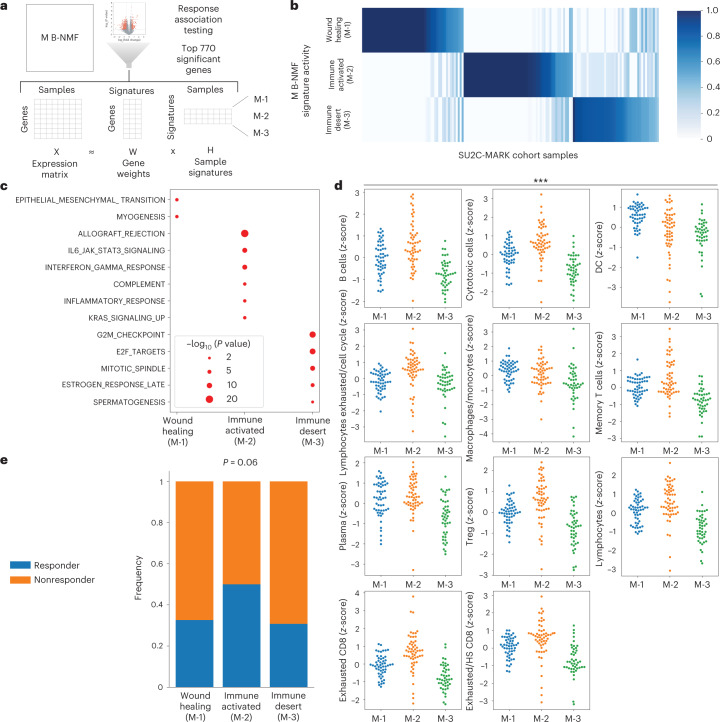
Derivation of M subtypes and association with checkpoint blockade response. **a**, Overview of M signature generation using B-NMF. **b**, H-matrix of SU2C-MARK samples and normalized M signature activity from semisupervised B-NMF. **c**, Dot plot of hallmark GSEA results for B-NMF-derived M signatures. Nominal *P* values from the one-sided hypergeometric test are shown. **d**, Swarmplots of selected tumor-associated immune cell signatures by M clusters. Myeloid cells were generally enriched in the wound healing (M-1, *n* = 52 RNA samples) subtype, while most immune cell types were enriched in the immune-activated (M-2, *n* = 56 RNA samples) subtype and depleted in the immune desert (M-3, *n* = 44 RNA samples) subtype (*P* < 0.001 for all signatures, Kruskal–Wallis test). **e**, Response rate by M subtype. The immune-activated (M-2) subtype was enriched for responders compared to the wound healing (M-1) and immune desert (M-3) subtypes (*P* = 0.06, one-sided Fisher’s exact test).

**Extended Data Fig. 7:**
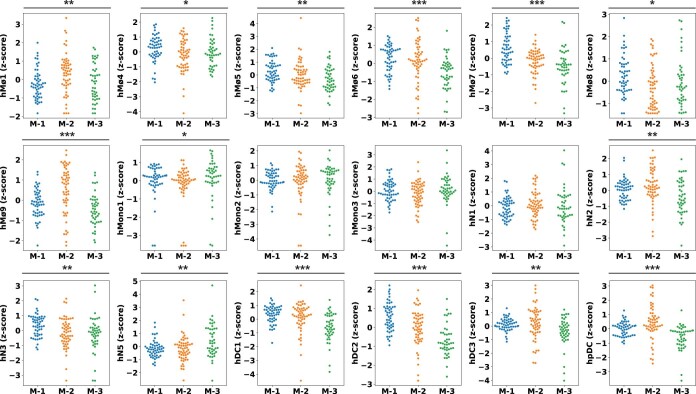
Comparison of single-cell profiling derived myeloid subsets with Microenvironment (M) subtypes in the SU2C-MARK Cohort. Swarmplot of myeloid cell signatures derived from single cell profiling^40^ across Microenvironmental subtypes. Significance of association was assessed by Kruskal–Wallis test (* p < 0.05, ** p < 0.01, *** p < 0.001).

To identify M signatures relevant to immunotherapy response beyond individual cell types, we applied Bayesian non-negative matrix factorization (B-NMF) to our top 770 differentially expressed genes, yielding three distinct M signatures as follows: M-1, M-2 and M-3 (Fig. 3a,b; Methods). Because these signatures were derived from bulk sequencing, they are expected to reflect the complete microenvironmental signature, inclusive of both tumor and nontumor (that is, immune and stromal) sources. GSEA of these signatures revealed M-1 to be associated with epithelial–mesenchymal transition (a gene set that includes wound healing and fibrosis) and M-2 to be associated with allograft rejection/IFN-γ response, consistent with an inflamed immune environment (Fig. 3c). M-3 had a weak association with cell cycle-related E2F targets, potentially reflecting a proliferative tumor signature, which in conjunction with the relative depletion of infiltrating myeloid and lymphoid cells, most resembles the previously reported immune desert phenotype^43^ (Fig. 3d and Extended Data Fig. 7). Notably, the response rate to checkpoint blockade varied across these subtypes, with increased response rates observed in M-2 relative to M-1 and M-3 (*P* = 0.06; Fig. 3e). Overall, these results suggest that there may be at least two distinct transcriptional states associated with checkpoint blockade resistance in NSCLC.

### Tumor intrinsic subtyping

**Fig. 4:**
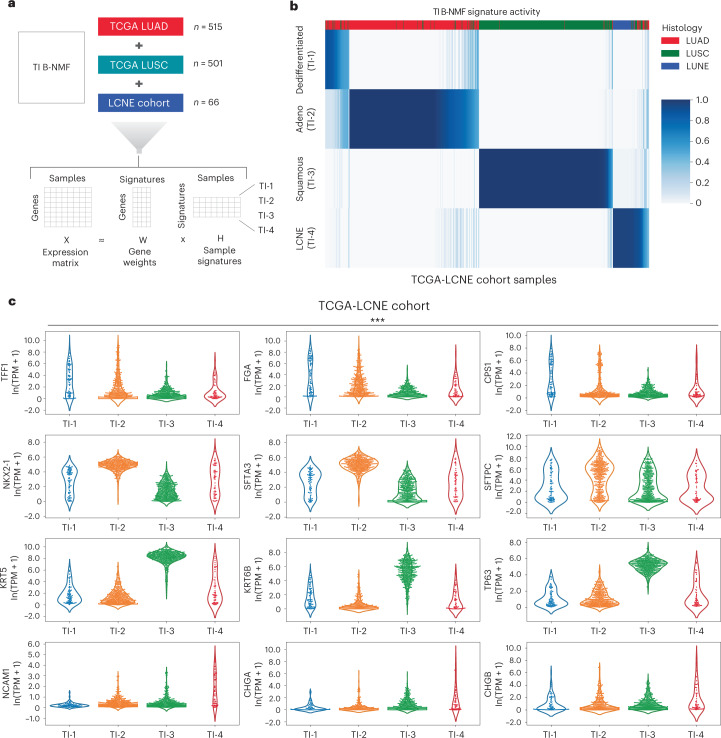
Derivation of TI NSCLC transcriptional subtypes. **a**, Overview of B-NMF approach to the generation of TI subtype signatures. A total of 1,082 RNA-seq samples spanning the three dominant NSCLC histologies were used as input for signature identification. Specifically, the TCGA LUAD and LUSC cohorts were used in addition to a published LCNE Cohort by George et al.^63^ to generate the combined TCGA-LCNE cohort. **b**, H-matrix of TCGA-LCNE samples and normalized TI signature activity. **c**, Violin plots of cancer subtype immunohistochemistry markers based on membership in TI clusters TI-1 (*n* = 81 samples), TI-2 (*n* = 433 samples), TI-3 (*n* = 447) and TI-4 (*n* = 55). Dedifferentiated (TI-1) samples expressed lower levels of canonical adenocarcinoma and squamous markers, but notably high levels of markers associated with neighboring endodermal lineages (top row). Significance was assessed by the Kruskal–Wallis test (***P < 0.001).

**Extended Data Fig. 8:**
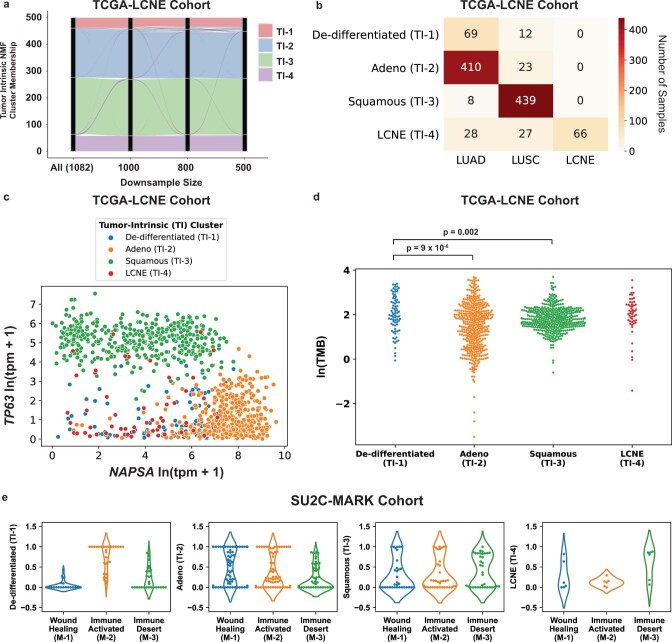
Extended analysis of Tumor-Intrinsic (TI) subtypes. **(a)** Alluvial plot of Tumor Intrinsic (TI) subtype downsampling analysis ranging from full TCGA-LCNE cohort (N = 1082) to under 50% downsample (N = 500). The TCGA-LCNE cohort comprises TCGA lung adenocarcinoma (LUAD), lung squamous cell carcinoma (LUSC), and published large cell neuroendocrine (LCNE)^63^ cohorts. Both overall distribution and individual sample membership were well preserved across downsamples. **(b)** Confusion matrix of TCGA-LCNE cohort comparing TI subtype assignment with study source. The novel de-differentiated (TI-1) subtype included predominantly TCGA LUAD samples, with a smaller contribution from TCGA LUSC. **(c)** Expression scatterplot of canonical adenocarcinoma and squamous cell carcinoma markers, *NAPSA* (Napsin A) and *TP63* (encoding both p40 and p63), respectively, across the TCGA-LCNE Cohort. Samples are colored by TI cluster assignment, with neither de-differentiated (TI-1) nor LCNE (TI-4) samples showing strong canonical lineage marker expression. TPM = transcripts per million. **(d)** Tumor mutation burden (TMB) for Tumor Intrinsic subtypes TI-1 (N = 81 patients), TI-2 (N = 433 patients), TI-3 (N = 447 patients), and TI-4 (N = 55 patients) in the TCGA-LCNE Cohort. The De-differentiated (TI-1) subtype had an increased mutation burden compared to the Adeno (TI-2) and Squamous (TI-3) subtypes (p = 9 ×10^−6^ and p = 0.002, respectively, two-sided Mann–Whitney U test). **(e)** Violinplots of Tumor Intrinsic signatures by membership in Microenvironment clusters M-1 (N = 52 RNA samples), M-2 (N = 56 RNA samples), and M-3 (N = 44 RNA samples).

Having explored aggregate microenvironmental states, we next turned our attention to tumor intrinsic expression factors that may have a relationship with response. To define relevant tumor intrinsic (TI) lung cancer subtypes, we assembled a large reference collection of over 1,000 transcriptomes (TCGA-LCNE) representing the three predominant NSCLC histologies, namely adenocarcinoma, squamous cell carcinoma and large cell neuroendocrine carcinoma (Fig. 4a and Methods). To define signatures of individual subtypes in this collection, we first performed B-NMF across this cohort, converging on a robust four-cluster solution (Fig. 4b and Extended Data Fig. 8a). Of these TI clusters, TI-1 and TI-2 contained predominantly adenocarcinomas, TI-3 was composed largely of squamous cell carcinomas, and TI-4 was primarily large cell neuroendocrine carcinomas (Extended Data Fig. 8b). Notably, unlike our M signatures above—which were derived solely from the subset of genes with significant response associations and were enriched for immune and stromal components—our TI signatures emerged from the unsupervised factorization of primary lung cohorts spanning three distinct histologies, explaining the high concordance between our TI subtypes and existing histologic categories.

To understand these signatures in more detail, we explored the expression of canonical markers of adenocarcinoma and squamous differentiation, namely *NAPSA* (which encodes Napsin A) and *TP63* (which encodes both p63 and p40), respectively (Extended Data Fig. 8c). While TI-2 and TI-3 showed the expected lineage marker preferences, TI-1 samples showed weak expression of both markers. Decreased expression of lung lineage markers has previously been described in a subtype of poorly differentiated adenocarcinomas in which markers for adjacent gut lineages (neighboring endodermal territories during development) can become activated^44^. Indeed, a comparison of these subtypes to immunohistochemical markers of various endodermal lineages revealed enrichment in these gut-specific marker genes in TI-1 samples, such as *TFF1*, *FGA* and *CPS1* (Fig. 4c). TI-1 samples were also notable for an elevated TMB relative to the well-differentiated TI-2 adenocarcinoma subtype and the TI-3 squamous subtype (Extended Data Fig. 8d).

**Fig. 5:**
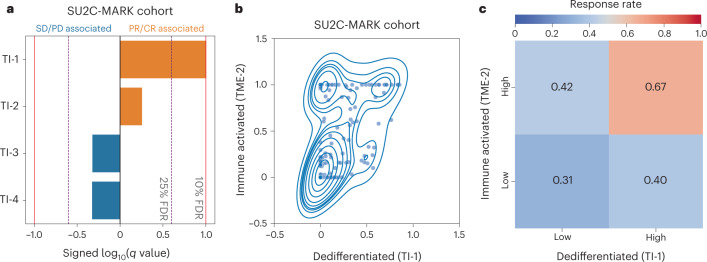
Association between TI signatures, M signatures and response in the SU2C-MARK cohort. **a**, Logistic regression analysis summary in the SU2C-MARK cohort between TI signatures and binned response category (PR/CR versus SD/PD). The dedifferentiated (TI-1) signature showed a significant association with response (*q* < 0.1, logistic regression with Benjamini–Hochberg adjustment). **b**, Kernel density estimate plot of the association between the activities of the dedifferentiated (TI-1) signature and the previously identified immune-activated (M-2) signature. **c**, Response rate in the SU2C-MARK cohort binned by expression of TI-1 and M-2 signatures. Patients with both high TI-1 and high M-2 show the highest response rate.

Having established a reference collection of TI expression signatures, we applied these signatures to RNA-seq data from the SU2C-MARK cohort and assessed their association with response to checkpoint inhibitors. Notably, the dedifferentiated TI-1 cluster was most closely associated with response (Fig. 5a), consistent with the elevated mutational burden in this subtype as well as its stronger association with the M-2 ‘immune-activated’ subtype (Fig. 5b and Extended Data Fig. 8e). Indeed, patients with both immune-activated (M-2) and dedifferentiation (TI-1) signatures had the highest response rates to checkpoint blockade (67% ORR; Fig. 5c). Thus, TI states and immune M signaling may independently and additively govern responses in NSCLC.

### Integrative cohort analysis

**Fig. 6:**
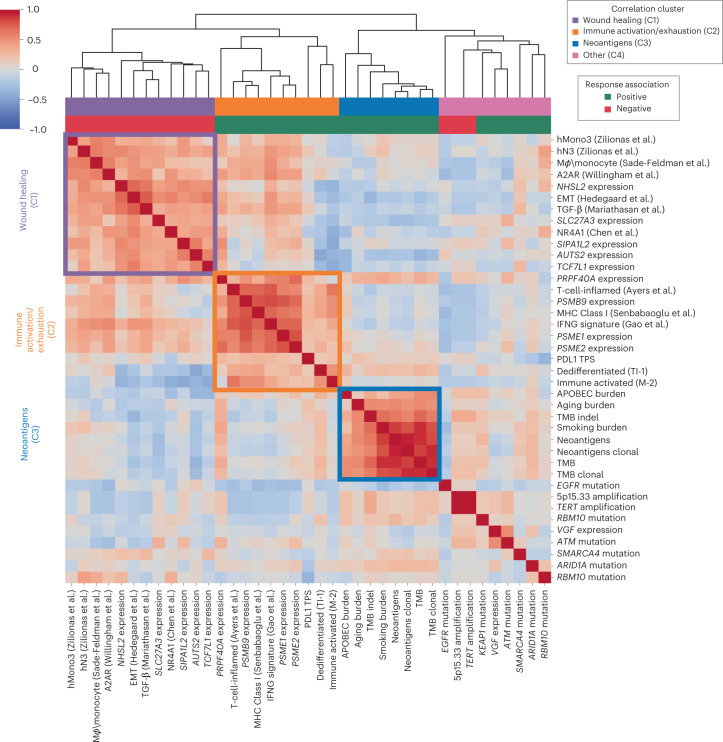
Clinical, genomic and transcriptomic feature integration across the SU2C-MARK cohort. Cross-correlation heatmap of the top response and resistance-associated features in the SU2C-MARK cohort along with a selection of signatures previously described as relevant to tumor and immune biology^35,45–50^. The three strongest correlation blocks are outlined and roughly correspond to wound healing (C1), immune activation/exhaustion (C2) and neoantigens (C3). Of note, the direction of association (that is, positive or negative) with immune checkpoint blockade response was consistent for predictors within each of these highlighted correlation blocks.

Having evaluated a broad set of clinical, genomic and transcriptomic features relevant to checkpoint blockade response in NSCLC, we set out to better understand the relationships between these predictors. Combining the top predictive features from each analysis, we generated a cross-correlation matrix to better understand how they relate to each other as well as to previously published signatures relevant to tumor biology and immune response (Fig. 6 and Methods)^35,45–50^. Notably, three strong correlation blocks could be observed, with consistent response associations within each subset. The first correlation block (C1) appeared to reflect a canonical ‘wound healing’ microenvironment, including immunosuppressive myeloid and stromal signatures. The second correlation block (C2) reflected the more classic cytokine and immune milieu associated with ‘immune activation/exhaustion,’ including both infiltrating immune signatures and proteasome subunits. The third correlation block (C3) consisted of features related to mutational burden, presumably all proxies for neoantigen abundance and consequent enhanced immune recognition.

**Extended Data Fig. 9:**
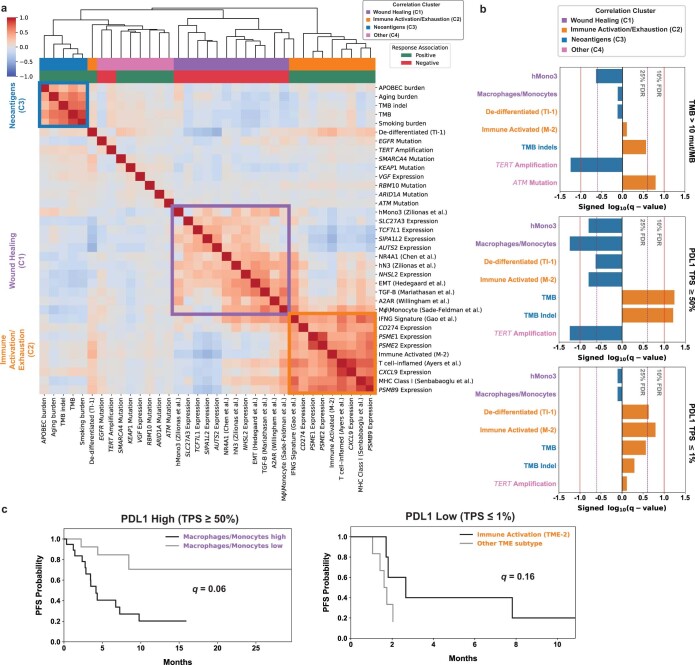
Evaluation of correlation in TCGA data between top SU2C-MARK predictors and assessment of their ability to further stratify clinically relevant subgroups of the SU2C-MARK cohort. **(a)** Cross-correlation heatmap of the top response and resistance associated features in the SU2C-MARK cohort as assessed in TCGA LUAD and LUSC combined datasets (N = 1018)^35,45–50^. Correlation cluster and response association colorbars based on designations in the SU2C-MARK cohort are plotted. Unsupervised hierarchical clustering re-identifies the previously recognized feature clusters corresponding to Wound Healing (C1), Immune Activation/Exhaustion (C2), and Neoantigens (C3). Nearly all features retain their original cluster designations (the relocation of the De-Differentiated TI-1 signature may relate to its association with high mutation burden as described earlier). **(b)** Contribution of SU2C-MARK predictors to clinically relevant biomarker subsets. The addition of features from the Wound Healing (C1) and Immune Activation/Exhaustion (C2) clusters meaningfully stratify traditionally favorable (for example, PDL1 high) and unfavorable (for example, PDL1 low) clinical subgroups (*q* = 0.06 and *q* = 0.16, respectively, Benjamini–Hochberg adjusted logrank test). TMB = Tumor Mutation Burden. **(c)** Association of top genomic predictors from SU2C-MARK cohort with Progression-Free Survival (PFS) for clinically relevant subgroups of NSCLC, namely high TMB ( > 10 mut/MB, top; favorable), high PD-L1 tumor proportion score (PDL1 TPS) corresponding to PDL1 TPS ≥ 50% (middle; favorable), and low PD-L1 expression (PDL1 TPS ≤ 1%, bottom; unfavorable). Signed FDR *q*-values based on Benjamini–Hochberg adjustment of logrank p-values are plotted for each feature (Methods).

The remaining nine features were somewhat loosely correlated as a fourth cluster (C4) enriched for single-gene alterations with potentially distinct immunobiologies. Notably, this cluster included *EGFR* mutations, which interestingly showed minimal association with the immune signatures but a moderate anticorrelation with mutational burden features, suggesting the intrinsic resistance of this subtype may predominantly be driven by insufficient neoantigens^15^ (Fig. 6 and Extended Data Fig. 9a).

To evaluate whether the additional genomic predictors identified in this study could augment existing biomarker-defined subsets of NSCLC, we selected the top two significant predictors from each cluster and evaluated their potential to further stratify progression-free survival (PFS) in three clinically relevant subgroups: TMB > 10 mut/MB (favorable; *n* = 27), PD-L1 TPS ≥ 50% (favorable; *n* = 34) and PD-L1 TPS ≤ 1% (unfavorable; *n* = 18). Following FDR correction, we identified multiple near-significant and significant associations (*q* < 0.25 and 0.1, respectively; Extended Data Fig. 9b,c and Methods), particularly when evaluating features from the immune activation/exhaustion and wound healing clusters (dedifferentiated TI-1 in PD-L1 TPS ≤ 1% *q* = 0.23; immune-activated M-2 in PD-L1 TPS ≤ 1% *q* = 0.16; macrophage/monocytes in PD-L1 TPS ≥ 50% *q* = 0.06; hMono3 in PD-L1 TPS ≥ 50% *q* = 0.11). Therefore, the presence of these factors may augment prediction based on standard clinical variables.

### Feature analysis in single-cell data

**Fig. 7:**
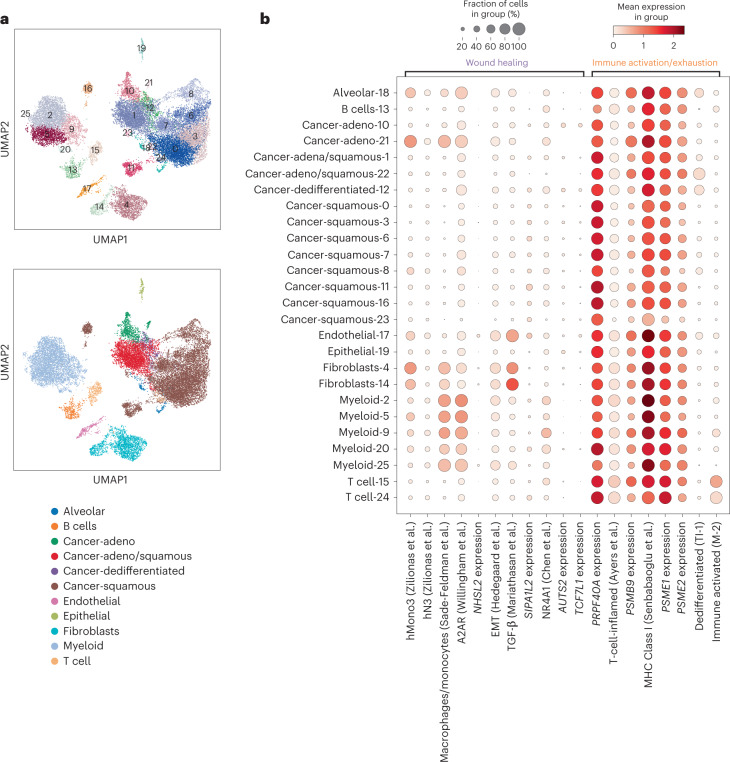
Exploration of top SU2C-MARK transcriptomic features in single-cell data. **a**, Leiden clustering of single-cell RNA-seq data from NSCLC^51^ colored by cluster ID (upper) or cell-type label (lower). Exploration of tumor markers within the cancer-specific clusters enabled further resolution into NSCLC subtypes, including recapitulation of the dedifferentiated TI-1 subtype identified earlier from bulk RNA-seq data (Cluster 12; Extended Data Fig. 8a). **b**, Association between cell types identified in NSCLC single-cell data and selected genes and metagenes from the wound healing (C1) and immune activation/exhaustion (C2) feature clusters in the SU2C-MARK cohort or with previously described relationships to immunotherapy response^35,45–50^. Features within larger correlation blocks in bulk RNA-seq data did not always arise from the same single-cell sources (for example, TGF-β versus macrophages/monocytes in the wound healing cluster, and dedifferentiated TI-1 versus immune-activated M-2 in the immune activation/exhaustion cluster).

**Extended Data Fig. 10:**
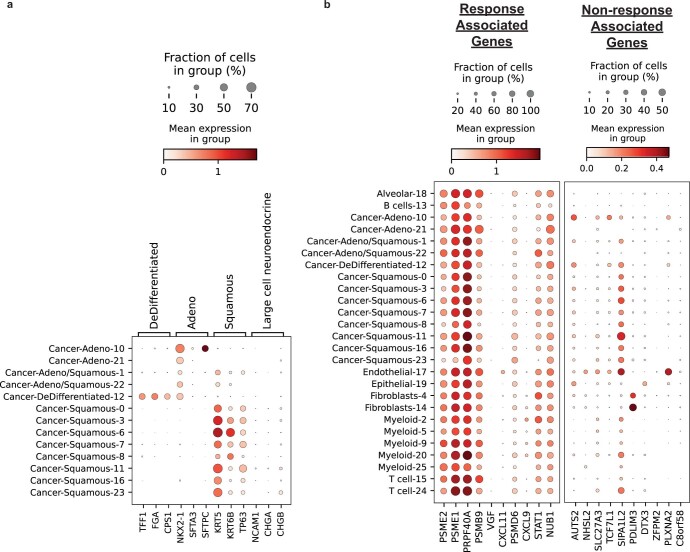
Cell type identification and feature analysis from previously published single cell RNA-Seq data in NSCLC. **(a)** Analysis of top immunohistochemistry (IHC) markers for Tumor Intrinsic (TI) subtypes in single cell non-small cell lung cancer (NSCLC) data^51^. Leiden cluster 12 demonstrated moderate expression of all 3 IHC markers for the De-Differentiated TI-1 subtype identified from bulk RNA-Seq. Other cluster demonstrated Adeno, Squamous, or mixed Adeno/Squamous markers, with no predominantly Large Cell clusters. **(b)** Dotplots of the top 10 favorable (left) and unfavorable (right) single genes identified in limma voom analysis of the SU2C-MARK cohort, as expressed in single cell NSCLC data. As observed for features in the larger correlation blocks earlier (Fig. 7b), individual predictors with uncorrelated single cell profiles could be found within each category (for example, *CXCL9* vs. *CXCL11* among favorable predictors, and *SIPA1L2* and *PDLIM3* within unfavorable predictors).

Given that the predictors identified in this study were derived from bulk specimens, they likely reflect contributions from multiple distinct cell types within the tumor microenvironment. To gain additional insight into the specific cellular components that may be driving response and resistance, we explored these predictors in the context of published single-cell sequencing data from NSCLC within mixed tumor environments that may be contributing to these signals in bulk data^51^. Evaluation of the marker expression from the 13 cancer-related clusters revealed a straightforward mapping to several TI subtypes described earlier, including one cluster (cluster 12) which mapped to our dedifferentiated TI-1 subtype (Fig. 7a and Extended Data Fig. 10a; Methods).

Deconvolution of the unfavorable wound healing (C1) predictors suggested that the EMT and TGF-β signatures predominantly reflected fibroblasts and endothelial cells as opposed to a mesenchymal epigenetic state per se within the tumor cells; conversely, some of the dominant single-gene transcriptional predictors such as *AUTS2* and *TCF7L1* demonstrated substantial tumor intrinsic expression (Fig. 7b and Extended Data Fig. 10b). Similarly, analysis of the favorable predictors in the immune activation/exhaustion cluster (C2) revealed that while immunoproteasome subunits are expressed in most cell types, CXCL9 may be predominantly expressed by myeloid sources, and CXCL11 may be primarily derived from endothelial cells (Fig. 7b and Extended Data Fig. 10b). Finally, our favorable dedifferentiated (TI-1) and immune-activated (M-2) predictors, while correlated at the bulk level, did appear to identify distinct subpopulations (cancer cells and T-cells, respectively) at the single-cell level, consistent with our labeling of these signatures as tumor intrinsic versus microenvironmental (Fig. 7b). Taken together, these findings suggest the presence of rich, interacting ecosystems that may broadly underlie response and resistance to checkpoint blockade and provide a collection of specific signaling pathways and cell types that may be promising targets for future intervention.

## Discussion

Comprehensive identification of predictors of checkpoint blockade response in patients with NSCLC has been limited by the availability of large, well-annotated patient cohorts with matched genomic data, particularly within individual cancer types. Here we present a joint analysis of the SU2C-MARK cohort, a collection of nearly 400 patients with NSCLC, enabling the identification of diverse molecular predictors of immunotherapy response.

Among the top genomic features identified were *ATM* mutation and *TERT* amplification. Given emerging literature associating *ATM* loss with the release of cytosolic DNA and activation of the cGAS/STING pathway in other cancer types^52–54^, it is conceivable that a similar mechanism underlies the association observed in our cohort between *ATM* loss and response. Although less well characterized in the context of immunotherapy, *TERT* amplification may serve a protective function against telomere crisis, thereby forestalling a parallel mechanism, which has been linked to cGAS/STING activation and subsequent sensitization to checkpoint blockade in mouse models^55^.

Transcriptomic analysis in the SU2C-MARK cohort was notable for the identification of immunoproteasome subunit genes as key predictors of response, with greater enrichment than general IFN-γ targets or proteasome subunits. These findings are consistent with those described in melanoma, where a supervised signature consisting specifically of *PSMB8* and *PSMB9* was found to be predictive of immune checkpoint blockade response^56^. We speculate that enhanced peptide supply to MHC-1 via increased expression of the PA28ɑβ complex and immunoproteasome may result in superior CD8^+^ T-cell responses. In addition, the altered cleavage specificity of the immunoproteasome relative to the constitutive proteasome—particularly in terms of preferences for branched-chain amino acids and chymotrypsin-like target sites^29^— may confer increased antigen *quality* in addition to quantity in immunoresponsive tumors.

Higher level organization of the strongest genes associated with response and resistance identified microenvironmental signatures previously associated with relevant immune states such as the immune-activated (M-2) signature and immune desert (M-3) signature. The wound healing (M-1) signature, although less well described in the context of lung cancer, does match the TGF-β transcriptional signature thought to drive T-cell exclusion in bladder cancer^35^. While the immune desert (M-3) signature was somewhat more enigmatic, the top-weighted genes appear to be largely tumor intrinsic, suggesting they may directly reflect a tumor state unfavorable to immune invasion. Consistent with this notion, one of the top-weighted genes in the signature, *DSC3*, is a component of intercellular desmosome junctions that can act as barriers to immune infiltration^57^.

In addition to features such as these global immune states that may have pan-cancer relevance, we also describe a dedifferentiated (TI-1) NSCLC-specific subtype identified independently in both bulk and single-cell data using unsupervised approaches. A similar subtype has been described in mouse lung cancer models featuring a decreased expression of classic lung lineage markers as well as enhanced expression of developmentally adjacent endodermal lineages^44^. The correlation between this tumor intrinsic state and our immune-activated (M-2) signature could represent an underlying differentiation state more susceptible to immune recognition (for example, via the presentation of oncofetal antigens)^58^, or conversely, a cell state change in response to an inflammatory cytokine milieu^59^. Establishing the direction of causality between these signatures may have important implications for further therapeutic intervention.

Finally, integrative analysis of our genomic features along with previously reported signatures relevant to immune and tumor biology supported the notion of a complex interplay between distinct signaling pathways (for example, CXCL9 versus TGF-β signaling) and distinct cell types (for example, myeloid cells versus fibroblasts), shedding light on some of the multifaceted interactions underlying checkpoint blockade responsiveness. Particularly noteworthy in this respect is the recognition that a number of features identified here may be truly tumor intrinsic predictors, which aside from a handful of specific driver events^15,16^ or defects in antigen presentation^26^ have been somewhat elusive in NSCLC. It is our hope that the SU2C-MARK cohort continues to serve as a rich resource for further unraveling the complex architecture of relevant genomic predictors, and for generating deeper insights into the biology of antitumor immunity.

## Methods

### Clinical cohort and assessment

All patients in the SU2C-MARK cohort consented through umbrella sequencing protocols approved under local institutional review board protocols at their respective cancer centers (Dana-Farber Cancer Institute 02-180, Massachusetts General Hospital 13-416, MD Anderson PA13-0589, Memorial Sloan Kettering 12-245, Columbia University IRB-AAA05706, University of California Davis LCRP-001, Yale 1411014879, Johns Hopkins IRB00100653). All samples in this study were from patients treated with anti-PD(L)1 therapy either as a single agent or in combination with other agents between 2009 and 2019. Although this cohort predominantly corresponds to standard-of-care therapy, a subset of patients from MSKCC treated with dual checkpoint blockade was derived from sequencing of specimens collected during the course of Checkmate 012 (NCT01454102; ref. ^64^).

Samples collected typically correspond to the first standard-of-care confirmation of metastatic disease, and therefore reflect a timepoint before receipt of any advanced therapy. Response data were assessed using RECIST v1.1 criteria through a dedicated radiologist review of standard-of-care clinical restaging studies (or in a subset of cases, imaging obtained while on a trial protocol). Confirmed BOR was determined using radiographic data following the first line of therapy involving a PD(L)1-based agent. PFS and overall survival were defined from the date of treatment start with a PD(L)1 agent until the first evidence of radiographic/clinical progression or date of death, respectively, and censoring was based on the date of last follow-up. To facilitate further analyses, WES and RNA-seq specimens were divided into two cohorts with cohort 1 corresponding to roughly the first 80% of available samples. Of note, a subset of these samples has been described previously in institution-specific collections^65,66^.

Informed consent was obtained under the institutional protocols listed above. Patients were not compensated for their participation. In all, the cohort consisted of 393 patients undergoing checkpoint blockade therapy. Patients in the cohort ranged in age from 29 to 90 years. In total 182 patients were male and 207 patients were female. Additional details on the cohort distribution are described in Extended Data Fig. 1a.

### WES

WES of DNA was performed at the Genomics Platform of the Broad Institute of Harvard and MIT as described previously^67,68^, with the exception of samples previously sequenced at Johns Hopkins^65^ and Yale University^69^. In brief, DNA was extracted from FFPE tumor specimens and either matched normal whole blood, or in cases where this was unavailable, from adjacent normal FFPE specimens. Extraction was performed using the Qiagen AllPrep DNA/RNA Mini Kit (80204). A single aliquot of 150–500 ng input DNA in 100 μl TE buffer was used for library generation. Library preparation was performed using the Kapa HyperPrep kit, and quantification was performed using PicoGreen. Adapter ligation was performed using the TruSeq DNA exome kit from Illumina per manufacturer’s instructions. Sequencing of pooled libraries was performed using a HiSeq2500 with 76 bp paired-end reads. The mean target coverages for tumor and normal samples were 150× and 80×, respectively.

### Somatic analysis of WES

Initial alignment of all samples to the hg19 genome was performed using the Broad Picard pipeline (v2.4.1), specifically with bwa 0.5.9 (ref. ^70^). The Broad Cancer Genome Analysis group somatic mutation pipeline was run in the cloud platform Firecloud/Terra. Specifically, the first-pass quality control was performed by assessing sample contamination using ContEst^71^ and identifying potential sample swaps using the Picard CrossCheckFingerprints tool (using software versions from the GATK 4.0.5.1 release). Somatic single nucleotide variants (SNVs) and indels were called using a combination of MuTect^72^, MuTect2 (ref. ^73^) and Strelka^74^. Recovery of somatic variants filtered due to tumor contamination in the matched normal was performed using DeTiN v1.7 (ref. ^75^) followed by annotation with Oncotator v1.9 (refs. ^75,76^). Adjacent SNV events were merged to di-nucleotide variants (DNVs), and filtering was performed using OxoG and FFPE Orientation Bias filters as well as removal of events observed in a panel of normals composed of TCGA and Illumina Capture Exome normals^77^. Finally, a BLAT realignment filter was implemented to eliminate potentially spurious variants resulting from mismapped reads^78^. To meet quality control criteria for inclusion in the exome cohort, samples were required to have mean and median target coverage >50×, contamination <5% and tumor purity >10% as assessed by ABSOLUTE (v1.5)^79^. Comparison of MutSig2CV^80^ driver analysis from the SU2C-MARK cohort agreed well with previously published results for TCGA lung adenocarcinoma (LUAD) and lung squamous cell carcinoma (LUSC) cohorts (Extended Data Fig. 2a).

### TMB and mutation signature analysis

TMB was calculated as the natural log of nonsynonymous SNVs, DNVs and indels in a sample divided by the size of the Illumina exome capture territory in megabases. Signatures for the SU2C-MARK cohort were determined using the SignatureAnalyzer Bayesian NMF (v1.2) method^81–83^. In brief, we pooled TCGA LUAD^21^, TCGA LUSC^20^ and SU2C-MARK cohort samples to improve our power for detection of rare signatures and performed unsupervised signature extraction using 20 random initializations. Thirteen runs converged to a seven-signature solution, so the *k* = 7 solution with maximum posterior probability was selected for downstream analysis. Assessment of cosine similarity between the seven signatures identified and the previously described COSMIC signatures^84^ was used to assign labels to each, with the three dominant signatures representing aging, APOBEC and smoking. Signature attributable mutation burden was calculated as the relative projection strength for each signature in a given sample. Dominant signatures identified across the cohort are shown in Extended Data Fig. 4. Log values of the mutation, signature and clonal/subclonal burdens were calculated using a pseudocount of one event per MB.

### Neoantigen analysis

Potential neoantigens were identified by first running POLYSOLVER (v1.0)^61^ to identify MHC Class I alleles from matched normal WES data. Predicted binding affinity for all possible 9mer and 10mer peptide sequences overlapping single and di-nucleotide somatic variants was assessed using NetMHCPan-4.0 (refs. ^60,85,86^). Neoantigens with percentile ranks of two or less for any Class I allele in the same patient were counted as predicted binders.

### Somatic copy number alteration analysis and GISTIC evaluation

Somatic copy number alterations were assessed from WES using the GATK4 CNV pipeline on Firecloud/Terra (corresponding to GATK v4.0.8.0). A copy number panel of normals (*n* = 820 samples) was generated from a collection of FFPE as well as fresh frozen samples filtered to have less than 1% of tumor in normal contamination. GATK CNV bin length was set to zero, and read counts were processed using the hg19 Illumina Capture Exome (ICE) targets with padding of 250 bases. Intervals were filtered for having less than 1% of samples with zero coverage by setting—maximum-zeros-in-interval-percentage to 1. The minimum total allele count for informative heterozygous SNPs was set to 10. GISTIC2.0 was used to process the allelic somatic copy number data to identify recurrent copy number altered regions across the cohort^87^. The continuous copy number output values (rather than binned value) for focal and gene-specific events from GISTIC were used as inputs for downstream analysis. Comparison of significant recurrent alterations showed good consistency between the SU2C-MARK cohort and prior TCGA publications (Extended Data Fig. 3).

### ABSOLUTE analysis

Tumor purity and ploidy were estimated using ABSOLUTE (v1.5)^79,88^. Specifically, somatic mutation and copy number data were used as inputs, and purity/ploidy solutions were evaluated manually. In general, solutions were selected with a preference for describing the observed data well at modeled integer copy numbers, being parsimonious (for example, diploid as opposed to genome doubled), appropriately fitting full deletions and having an alpha/2 line centered within the highest somatic SNV allelic fraction peak (where alpha represents the model purity). A gene-specific integer copy number and LOH were inferred from integer copy number segmented output from total or allele-specific copy number analysis, respectively. Samples with less than 10% purity were excluded as a filtering step during WES quality control as above.

### Subclone evaluation using PhylogicNDT

The subclonal architecture was inferred from ABSOLUTE input using PhylogicNDT (v1.0)^62^. Mutation clonality across single samples was modeled using a Dirichlet process, enabling the assignment of mutations to discrete subclones with imputed cancer cell fractions (CCFs). Variants assigned to clusters with CCF over 0.85 were classified as clonal, while the remainder were deemed subclonal. Subclone count was based on the total number of unique subclones identified by 1D Phylogic analysis.

### T-cell and B-cell infiltrate analysis

Rearranged reads corresponding to T- and B-cell receptors were identified from WES data using MiXCR v3.0 (ref. ^27^). Primary BAM files were processed with the ‘analyze shotgun’ pipeline, and reads corresponding to TCR or Ig clonotypes with productive rearrangements (that is, those leading to in-frame rearrangements without stop codons) were summed to give a total TCR or Ig read count per sample. To infer relative T- or B-cell abundance, these read counts were normalized by calculating T-cell and B-cell burden^89^, defined as (rearranged receptor count reads + 1)/(aligned reads/10^6^). Natural log of this burden metric was used during the significance assessment.

### Response association testing

In total, 106 features derived from whole exome and transcriptome analysis were evaluated (Supplementary Table 30). Features reflecting mutation burden (for example, TMB, Neoantigens, etc.) were log-transformed before evaluation. Mutation and copy number features were filtered to include only those present in at least 5% of the cohort. Each feature was assessed in a univariate logistic regression model of BOR, binned as responders (PR/CR) versus nonresponders (SD/PD). FDR calculation was performed using the Benjamini–Hochberg method, with features categorized as significant (FDR < 0.1) or near-significant (FDR < 0.25).

### Whole transcriptome sequencing

RNA-seq data were processed using the GTEx RNA-seq pipeline^90^ with the use of the GENCODE v19 reference transcriptome, followed by quality control evaluation using the RNA-SeQC2 (v1.0) pipeline^90,91^, generating both expression data as transcripts per million (TPM) as well as quality metrics. Specifically, this pipeline uses STAR (v1.0) alignment with the following settings: alignIntronMax = 1,000,000, alignIntronMin = 20, alignMatesGapMax = 1,000,000, alignSJDBoverhangMin = 1, alignSoftClipAtReferenceEnds = True, chimJunctionOverhangMin = 15, chimMainSegmentMultNmax = 1, chimSegmentMin = 15. Alignment is then followed by: (1) omission of reads that are unmapped, have secondary alignments (0 × 100 flag) or have the quality control fail flag (0 × 200), and (2) filtering for high-quality exonic reads that uniquely map as pairs (0 × 2 flag) and have fewer than six mismatches to ultimately generate gene-level expression data as well as associated quality metrics. Using the median exon TPM (CV), the number of genes detected, and other measures, we selected the highest quality samples (*n* = 152) for subsequent analysis.

### RNA-seq differential expression analysis

To analyze differentially expressed genes, we restricted our search to protein-coding transcripts, and those minimally expressed at a log_2_TPM of 0.5 or higher in at least 30% of our samples. Using the BOR groupings of responders (PR/CR) versus nonresponders (SD/PD), we then used the R package limma voom to identify genes differentially expressed with respect to response.

### Gene set enrichment analysis

Using the signed, log-transformed *P* values from the differential expression results, we performed enrichment analyses using the ‘fgsea’ package (v3.16)^92^ and the Hallmark Gene Sets from the Molecular Signatures Database (MSigDB)^33^.

### RNA-seq supervised signature analysis

Using existing literature, we derived metagenes for clinically important features. Starting with groups of genes associated with a certain feature (for example, genes expressed according to B-cell abundance), we took the mean of the log_2_-transformed TPMs in our cohort, then compared samples to each other by *z*-scoring those averages. These analyses include metagenes for different groups of leukocytes^93^, which we use as a proxy for the level of immune infiltration indicated by RNA-seq. Additionally, we used previously published markers of developmental lineage^44,94^ and NSCLC subtypes^95^ to better understand the developmental identity of each sample. We also defined an additional gene set for neuroendocrine identity using markers from a published characterization of large-cell neuroendocrine lung cancer^63^. For cell type-specific characterization, we used metagenes from single-cell studies of lung cancer developmental subtypes and immune infiltrate^37,40^.

### Non-negative matrix factorization-based expression subtyping

We applied the B-NMF algorithm^81,89,96,97^ to organize the significantly differentially expressed gene set from cohort 1 of our RNA-seq data (*n* = 123) into three distinct clusters, that is, our M subtypes. We first filtered our log_2_(TPM + 1) gene expression matrix to keep only genes with differential expression *P* value < 0.05 and absolute log-fold change > 0.5, thus limiting our analysis to genes potentially involved in response. We further filtered out genes with sparse or low expression, that is more than 10% NA or zero values, or in the bottom 10% of mean expression. We transformed the values to fold changes by subtracting the median for each gene, then obtained the Spearman correlation matrix of these fold changes, and performed hierarchical clustering while varying the number of clusters (K) from 2 to 10 and repeating 500 iterations for each *K* value. We then obtained consensus matrices for each *K* (calculating the number of times samples clustered together in the 500 iterations), summed these matrices across all *K* values, and normalized the resulting matrix by the number of iterations. Using B-NMF with a half-normal prior, this matrix was used to decide on the optimal number of clusters. Using this empirically determined value of *K*, we then applied the B-NMF algorithm to the original log_2_TPM gene expression matrix. In this case, the gene expression matrix is approximated by W*H, where *H* is the cluster membership matrix and *W* is the gene weight matrix. We used the *W* matrix to narrow down the genes most closely associated with each cluster, keeping only genes in the top 50% of normalized weights for each cluster, as well as those with the largest difference between within-cluster versus outside-cluster expression. Using this reduced marker gene list, we classified the remaining samples in cohort 2 into our three-cluster scheme. Of note, given re-annotation of the RNA sample from patient SU2CLC-DFC-DF0732 as having been post-treatment, this specimen was removed from our analysis (Supplementary Note and Supplementary Fig. 1). We used this same procedure to define the TI subtypes, with the exception of initially filtering to keep high-variance genes (instead of keeping genes of interest from the differential expression analysis, as in the M subtypes). We similarly used TI marker genes to classify additional samples.

### Integrative predictor clustering

A collection of the top clinical, genomic and transcriptomic predictors identified in the SU2C-MARK cohort or published previously as relevant to antitumor immunity were first compared across samples in the SU2C-MARK cohort. Unsupervised hierarchical clustering was performed on this set, identifying four broad clusters that were ultimately designated wound healing, immune activation/exhaustion, neoantigens and others. As validation of these predictor classes, recalculation of these features was performed in TCGA data by combining publicly available mutation calling and RNA-seq data for the TCGA LUAD^21^ and TCGA LUSC^20^ cohorts (combined *n* = 1018). Unsupervised hierarchical clustering was again performed, and feature membership was compared to assignments made earlier from analysis within the SU2C-MARK dataset. As with other sections described here, integrative analysis was performed with Python (v3.7) and R (v3.4).

### Single-cell analysis of predictor clusters

Using single-cell data from a previously published NSCLC cohort^51^, we performed preprocessing, integration and Leiden clustering in Scanpy (v1.9.1)^98^ to identify distinct cell types. For preprocessing, we filtered counts to cells with at least 200 genes, and then filtered out genes that were observed in fewer than 50 cells. Further filtering was performed on cells with between 1,000 and 8,000 genes, total counts between 3,000 and 100,000, percent of mitochondrial counts less than 15%, and percent of ribosome counts less than 20%. Cell cycle effects were regressed out using Scanpy, and samples were then integrated using Harmony (harmony2019)^99^. The cell type of the Leiden clusters was annotated based on gene markers described in Wu et al.^51^ as well as canonical IHC cancer subtype markers. Clusters were assigned one of the cell types alveolar, B cell, cancer, endothelial, epithelial, fibroblasts, myeloid or T cell based on these expression markers. Metagene expression level was calculated as the mean expression of the gene markers that comprised the metagene. For signatures M-2 and TI-1, the top ten genes by weight were selected. Of note, in some cases, single genes or individual genes in a signature did not pass filtering or were not detected, and therefore were not plotted/included in a given metagene.

### Survival analysis

Single-feature survival analysis was performed using progression-free and overall survival data with censoring as described above. For the MSK impact cohort, patients with alterations in *ATM* found on panel sequencing who also had received checkpoint blockade therapy were included in the cohort. For integrative analysis across the feature list, the top two genomic features from each correlation cluster were selected for PFS analysis as follows: the monocyte/macrophage score, the hMono3 score, dedifferentiated signature TI-1, immune-activated signature M-2, TMB, TMB indel, *ATM* Mutation and *TERT* amplification. Participants were binned into high and low categories for each feature (using 0 as a cut point for *z*-score features, cluster identity for signatures, median for mutation burden features and the presence or absence of alteration/copy gain for single-gene features). FDR values were subsequently computed from the nominal *P* values obtained via the log-rank test using the Benjamini–Hochberg method. A complete list of the log-rank test results including median PFS for each subgroup is provided in Supplementary Table 31.

### Statistics and reproducibility

This study was designed as a retrospective immunogenomic analysis of biospecimens from NSCLC patients receiving checkpoint blockade in the advanced setting. As such, no statistical method was used to predetermine the sample size. Patients who did not have at least one pretreatment whole exome or RNA-seq sample that passed QC following library construction or alignment were excluded from the analysis (as described above). There was no randomization or stratification performed during described analyses, and investigators were not blinded to participant outcomes during primary data analysis.

### Reporting summary

Further information on research design is available in the Nature Portfolio Reporting Summary linked to this article.

## Online content

Any methods, additional references, Nature Portfolio reporting summaries, source data, extended data, supplementary information, acknowledgements, peer review information; details of author contributions and competing interests; and statements of data and code availability are available at 10.1038/s41588-023-01355-5.

## Supplementary information

Supplementary Information Supplementary Note and Supplementary Fig. 1.

Reporting Summary

Supplementary Tables Supplementary Tables 1–31.

## Data Availability

Raw sequencing data for WES and RNA-seq specimens in the SU2C-MARK cohort are available in dbGaP (phs002822.v1.p1), except for samples from Cleveland Clinic and UC Davis, as these sites did not explicitly include language around deposition of identifiable data in a controlled access repository. Further information about these collections can be obtained from the respective IRB teams (irb@ccf.org and hs-irbeducation@ucdavis.edu) and/or the PIs at each institution (UC Davis; PI: Riess – jwriess@ucdavis.edu; Cleveland Clinic; PI: Pennell – penneln@ccf.org). Data use restrictions specific to each site are also enumerated in the dbGaP accession and include Disease-Specific use (Dana-Farber Cancer Institute), Health/Medical/Biomedical use (MDA Anderson, Memorial Sloan Kettering), and General Research Use (Massachusetts General Hospital). Data from institution-specific cohorts is currently available in dbGaP under accession codes phs001618.v1.p1 (ref. ^66^) and phs001940.v2.p1 (ref. ^65^) as well as European Genome-phenome Archive EGAS00001003892 (ref. ^65^).

## Code availability

Code generated for this study has been deposited in the linked Zenodo repository: 10.5281/zenodo.7625517 (ref. ^100^).
