# Supplementary material for: Genomic and transcriptomic analysis of checkpoint blockade response in advanced non-small cell lung cancer
Source: Nat Genet. 2023 Apr 6;55(5):807–19. doi: 10.1038/s41588-023-01355-5 (PMC10181943; doi:10.1038/s41588-023-01355-5)
Supplement: Supplementary file 1 — Supplementary Note and Supplementary Fig. 1. [file 41588_2023_1355_MOESM1_ESM.pdf]

nature genetics

Article https://doi.org/10.1038/s41588-023-01355-5

Genomic and transcriptomic analysis of checkpoint blockade response in advanced non-small cell lung cancer

In the format provided by the authors and unedited

Supplementary Information

Supplementary Figures

Supplementary Figure 1. Significance analysis for SU2C-MARK cohort before and after sample exclusion. 2

Supplementary Notes

Supplementary Note 1. Brief note on quality control following sample exclusion. 4

Supplementary Figure 1. Significance analysis for SU2C-MARK cohort before and after sample exclusion.

Comparison of $-\log_{10}$(q-value) following False Discovery Rate (FDR) correction for all 106 features in the SU2C-MARK cohort before and after exclusion of RNA sample SU2CLC-DFC-DF0732. FDR q-values before and after exclusion are denoted as q_1 and q_2, respectively. Features that retained significance following sample exclusion are displayed in orange, and features that remained non-significant are displayed in blue. No features transitioned between significant and non-significant categories.

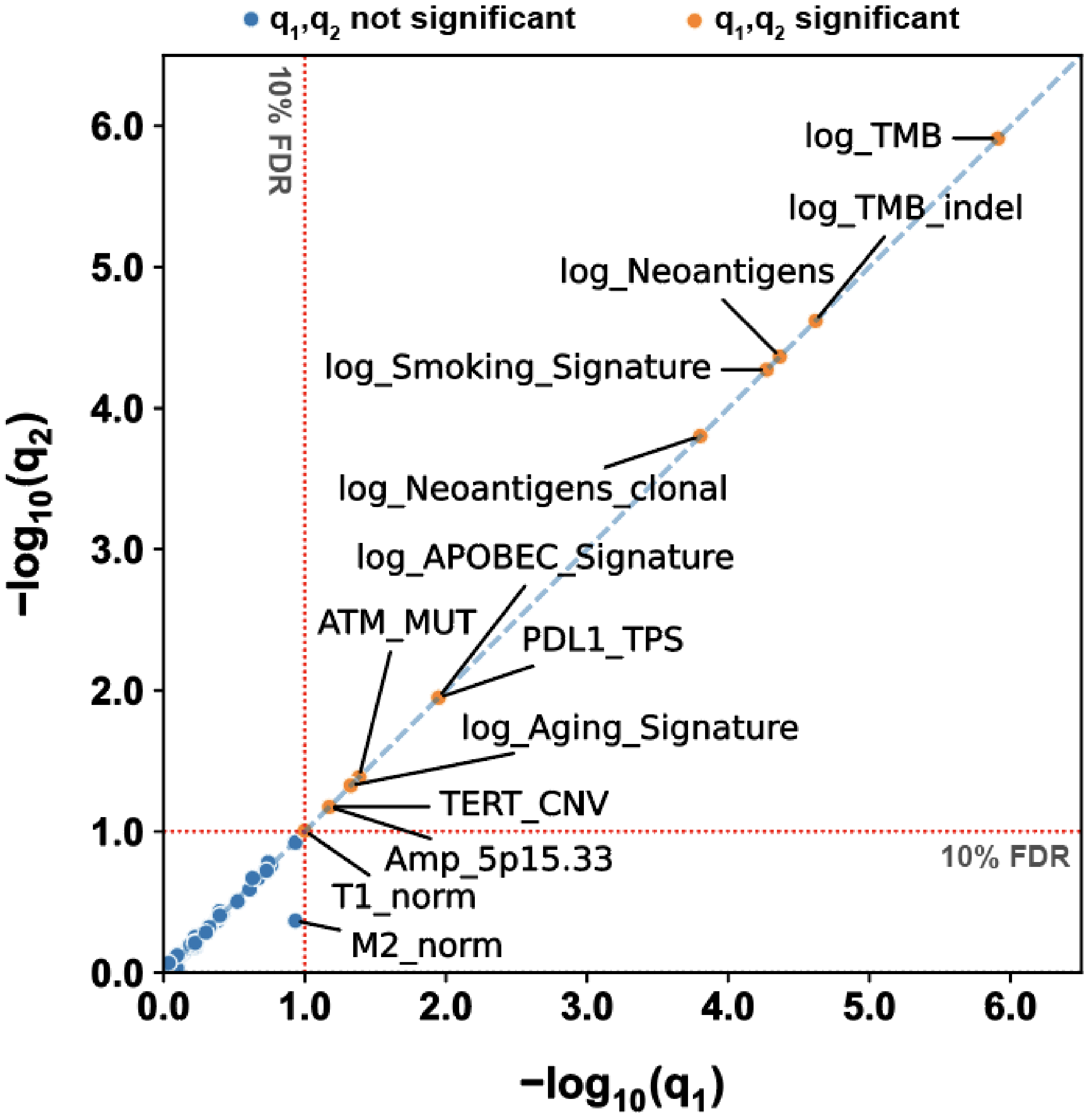

q1,q2 not significant
q1,q2 significant
10% FDR
log_TMB
log_TMB_indel
log_Neoantigens
log_Smoking_Signature
log_Neoantigens_clonal
log_APOBEC_Signature
ATM_MUT
PDL1_TPS
log_Aging_Signature
TERT_CNV
Amp_5p15.33
T1_norm
M2_norm
10% FDR
−log10(q2)
−log10(q1)
0.0
1.0
2.0
3.0
4.0
5.0
6.0

Supplementary Note 1. Brief note on quality control following sample exclusion.

Following re-annotation of one of our RNA-Seq samples (SU2CLC-DFC-DF0732) as a post-treatment rather than pre-treatment specimen, we compared the significance values of the feature list before and after exclusion of this specimen. As shown in **Supplementary Figure 1**, False Discovery Rate (FDR) q-values across the cohort were essentially unchanged, and no features transitioned from significant to non-significant or vice versa demonstrating the robustness of the analysis. Among the features, the largest impact was observed for "M2_norm," consistent with the fact that SU2CLC-DFC-DF0732 had high M2 signature activity.
