# Supplementary material for: Genomic and transcriptomic analysis of checkpoint blockade response in advanced non-small cell lung cancer
Source: Nat Genet. 2023 Apr 6;55(5):807–19. doi: 10.1038/s41588-023-01355-5 (PMC10181943; doi:10.1038/s41588-023-01355-5)
Supplement: Supplementary file 2 — Reporting Summary [file 41588_2023_1355_MOESM2_ESM.pdf]

nature portfolio

Corresponding author(s): Justin Gainor, Nir Hacohen, Gad Getz

Last updated by author(s): Dec 1, 2022

Reporting Summary

Nature Portfolio wishes to improve the reproducibility of the work that we publish. This form provides structure for consistency and transparency in reporting. For further information on Nature Portfolio policies, see our Editorial Policies and the Editorial Policy Checklist.

Statistics

For all statistical analyses, confirm that the following items are present in the figure legend, table legend, main text, or Methods section.

n/a	Confirmed	
☐	☒	The exact sample size (*n*) for each experimental group/condition, given as a discrete number and unit of measurement
☐	☒	A statement on whether measurements were taken from distinct samples or whether the same sample was measured repeatedly
☐	☒	The statistical test(s) used AND whether they are one- or two-sided<br>*Only common tests should be described solely by name; describe more complex techniques in the Methods section.*
☐	☒	A description of all covariates tested
☐	☒	A description of any assumptions or corrections, such as tests of normality and adjustment for multiple comparisons
☐	☒	A full description of the statistical parameters including central tendency (e.g. means) or other basic estimates (e.g. regression coefficient) AND variation (e.g. standard deviation) or associated estimates of uncertainty (e.g. confidence intervals)
☐	☒	For null hypothesis testing, the test statistic (e.g. *F*, *t*, *r*) with confidence intervals, effect sizes, degrees of freedom and *P* value noted<br>*Give P values as exact values whenever suitable.*
☒	☐	For Bayesian analysis, information on the choice of priors and Markov chain Monte Carlo settings
☐	☒	For hierarchical and complex designs, identification of the appropriate level for tests and full reporting of outcomes
☐	☒	Estimates of effect sizes (e.g. Cohen's *d*, Pearson's *r*), indicating how they were calculated

Our web collection on statistics for biologists contains articles on many of the points above.

Software and code

Policy information about availability of computer code

Data collection	No special software was used for clinical data collection
Data analysis	Exome data was aligned to hg19 using bwa 0.5.9 using the Terra platform. The Broad Picard pipeline (v2.4.1) was used for initial quality metrics (including ContEst, CrossCheckFingerprints), followed by mutation calling via MuTect1, Mutect2, and Strelka, recovery of variants using DeTiN v1.7, annotation of variants using Oncotator v1.9, filtering using OxoG and FFPE Orientation Bias filters as well as a BLAT realignment filter corresponding to GATK 4 pipeline (v4.0.5.1). ABSOLUTE v1.5, Phylogic-NDT (v1.0) and MutSig (MutSig2CV) were then applied in order to analyze the mutation data. Mutation signatures were generated using Mutation Signature Analyzer v1.2. Neoantigens were assessed using POLYSOLVER (v1.0) and NetMHCPan-4.0. Somatic copy number analysis was performed using GATK CNV (corresponding to GATK v4.0.8.0) followed by significance testing in GISTIC 2.0. Immune receptor abundance was inferred via MiXCR v3.0. Whole transcriptome sequencing analysis was performed using the GTEx RNA-Seq pipeline with GENCODE v19 annotation. Specifically STAR (v1.0) alignment was performed followed by quantification using RNA-SeQC2 (v1.0). Differential expression analysis was performed using the Limma-Voom package. Gene set enrichment was performed using 'fgsea' (v3.16) as well as the Molecular Signatures Database (MSigDB). Single cell analysis was performed using Scanpy (v1.9.1) and Harmony (harmony2019). Figure generation was performed in Python (v3.7) and R (v3.4).

For manuscripts utilizing custom algorithms or software that are central to the research but not yet described in published literature, software must be made available to editors and reviewers. We strongly encourage code deposition in a community repository (e.g. GitHub). See the Nature Portfolio guidelines for submitting code & software for further information.

Data

Policy information about availability of data

All manuscripts must include a data availability statement. This statement should provide the following information, where applicable:

- Accession codes, unique identifiers, or web links for publicly available datasets
- A description of any restrictions on data availability
- For clinical datasets or third party data, please ensure that the statement adheres to our policy

Raw sequencing data for WES and RNA-Seq specimens in the SU2C-MARK cohort will be available in dbGaP upon publication (phs002822.v1.p1), except in cases where consent was deemed not consistent with deposition in a controlled access repository (Cleveland Clinic, UC Davis). Data use restrictions specific to each site are also enumerated in the dbGaP accession and include: Disease-Specific use (Dana-Farber Cancer Institute), Health/Medical/Biomedical use (MDA Anderson, Memorial Sloan Kettering), and General Research Use (Massachusetts General Hospital). Data from institution specific cohorts is currently available in dbGaP under accession codes phs001618.v1.p169 and phs001940.v2.p1 as well as European Genome-phenome Archive EGAS0000100389268.

Field-specific reporting

Please select the one below that is the best fit for your research. If you are not sure, read the appropriate sections before making your selection.

☒ Life sciences ☐ Behavioural & social sciences ☐ Ecological, evolutionary & environmental sciences

For a reference copy of the document with all sections, see nature.com/documents/nr-reporting-summary-flat.pdf

Life sciences study design

All studies must disclose on these points even when the disclosure is negative.

Sample size

N = 393. This study was designed as a retrospective immunogenomic analysis of biospecimens from NSCLC patients receiving checkpoint blockade in the advanced setting. As such, no statistical method was used to predetermine the sample size. Patients who did not have at least one pre-treatment whole exome or RNA-Seq sample that passed QC following library construction or alignment were excluded from the analysis.

Data exclusions

No exclusions for available clinical data (though there was some missingness). QC was performed for whole exome and RNA-seq data as described in the Methods.

Replication

Reproduction of the central integrative clusters of the paper was attempted using whole exome and RNA-Seq data from TCGA, and demonstrated successful replication of the 4 feature clusters identified in this analysis (Extended Data Figure 9a).

Randomization

Given that this was not an interventional study, we did not randomize our participants but rather analyzed data as a single, unified cohort.

Blinding

Given that this was not an interventional study, we did not perform blinding in our analysis.

Reporting for specific materials, systems and methods

We require information from authors about some types of materials, experimental systems and methods used in many studies. Here, indicate whether each material, system or method listed is relevant to your study. If you are not sure if a list item applies to your research, read the appropriate section before selecting a response.

Materials & experimental systems

n/a	Involved in the study
☒	☐ Antibodies
☒	☐ Eukaryotic cell lines
☒	☐ Palaeontology and archaeology
☒	☐ Animals and other organisms
☐	☒ Human research participants
☐	☒ Clinical data
☒	☐ Dual use research of concern

Methods

n/a	Involved in the study
☒	☐ ChIP-seq
☒	☐ Flow cytometry
☒	☐ MRI-based neuroimaging

Human research participants

Policy information about studies involving human research participants

Population characteristics

This cohort represents a collection of 393 patients undergoing checkpoint blockade therapy for advanced NSCLC. Patients in

Population characteristics	the cohort ranged in age from 29-90. 182 patients were male, and 207 patients were female. Additional details on the cohort distribution are described in Extended Data Table 1.
Recruitment	Patients were consented during standard of care treatment (with the exception of a subset of patients consented as part of Checkmate 012/NCT01454102). Given the potentially longer timeframe in which responding patients were potentially consentable as well as the selection towards academic cancer centers, it is possible that this study has a bias towards patients with improved outcomes on checkpoint blockade as well as a sociodemographic tilt away from traditionally under-represented groups in medical research.
Ethics oversight	(Dana-Farber Cancer Institute #02-180, Massachusetts General Hospital #13-416, MD Anderson #PA13-0589, Memorial Sloan Kettering #12-245, Columbia University #IRB-AAA05706, University of California Davis #LCRP-001, Yale #1411014879, Johns Hopkins #IRB00100653)

Note that full information on the approval of the study protocol must also be provided in the manuscript.

Clinical data

Policy information about clinical studies

All manuscripts should comply with the ICMJE guidelines for publication of clinical research and a completed CONSORT checklist must be included with all submissions.

Clinical trial registration	Patients were obtained from retrospective standard of care cohorts with appropriate consent as outlined in our methods with the exception of patients from Checkmate 012/NCT01454102.
Study protocol	https://clinicaltrials.gov/ct2/show/NCT01454102
Data collection	Results correspond to a multi-arm Phase I study. Study sites were UCLA, Yale, Moffitt, Johns Hopkins, MSK, Duke, Fox Chase, UT Southwestern, University of Washington, and 3 Canadian health systems (Hamilton, Ottawa, Toronto). The study was conducted from 12/16/11 to 7/23/21.
Outcomes	Our analysis involved inclusion of previously sequenced biospecimens from this trial (rather than participation in any components of the design or analysis of the previously completed and published study).
